# Chimerism and population dieback alter genetic inference related to invasion pathways and connectivity of biofouling populations on artificial substrata

**DOI:** 10.1002/ece3.4817

**Published:** 2019-02-21

**Authors:** Ashleigh Marie Watts, Grant A. Hopkins, Sharyn Jane Goldstien

**Affiliations:** ^1^ School of Biological Sciences University of Canterbury Canterbury New Zealand; ^2^ Coastal and Freshwater Group Cawthron Institute Nelson New Zealand; ^3^ Present address: Tonkin + Taylor International Ltd Christchurch New Zealand

**Keywords:** chimerism, *Didemnum vexillum*, nonindigenous species, population connectivity, winter regression

## Abstract

Disentangling pathways by which nonindigenous species expand and spread regionally remains challenging. Molecular ecology tools are often employed to determine the origins and spread of introduced species, but the complexities of some organisms may be reducing the efficacy of these tools. Some colonial species exhibit complexities by way of chimerism and winter colony regression, which may alter the genetic diversity of populations and mask the connectivity occurring among them. This study uses nuclear microsatellite data and simple GIS‐based modeling to investigate the influence of chimerism and winter regression on the genetic diversity and patterns of genetic population connectivity among colonies of *Didemnum vexillum* on artificial substrates. Colonies sampled in summer were shown to form a metapopulation, with high levels of admixture, extreme outcrossing, and some substructure. These patterns were consistent within the subsampled winter colonies and with the inclusion of chimeric data. However, allelic richness and diversity were significantly different between winter and summer samples, altering interpretations relating to population connectivity and pelagic larval duration. This study demonstrates the importance of including seasonal sampling and imperative life history traits in genetic studies for clear interpretations and the successful management of introduced species.

## 1 INTRODUCTION

Nonindigenous biofouling communities now dominate many artificial structures in harbours and estuaries around the world, prompting investigations to understand and limit the invasiveness of these introduced communities. To this end, numerous studies have employed genetic markers to identify the extent of introductory pathways, the role of natural and anthropogenic vectors, and the rate of postborder spread (Goldstien, Schiel, & Gemmell, 2010; Pérez‐Portela, Turon, & Bishop, 2012; Zhan et al., 2012). However, much like the field of biogeography and studies of natural dispersal, the life history of marine species is often overlooked or is peripheral to studies applying population genetic tools (Ben‐Shlomo, 2017; Zhu, Degnan, Goldstien, & Eldon, 2015). More recently, genomic studies are revealing diversity in functional genes that may contribute to rapid evolution or enhanced competitiveness for introduced species (Papacostas et al., 2017; Smith, Abbott, Saito, & Fidler, 2015). The information gained from these genomic studies may lead to a better understanding of the nonindigenous communities, and, therefore, more effective management tools. However, the incorporation of such information into the identification of pathways and spread of nonindigenous species (NIS) remains unclear, when we are yet to incorporate known phenomena, such as the stochasticity and complexity of broadcast spawning and fragmentation (Eldon & Wakeley, 2009; Zhu et al., 2015), increased genetic diversity due to chimerism (Ben‐Shlomo, 2017), and the seasonality of occurrence (Fletcher, Forrest, Atalah, & Bell, 2013).

Chimerism is particularly problematic for genetic studies, but is yet to be comprehensively investigated and incorporated into analyses and interpretations. In a chimeric colony, two copies of a gene can occur in a single zooid, resulting in multiple copies within a small section of the colony tissue. Multiple studies have now identified chimerism in NIS, such as the colonial Botryllid and Didemnid ascidians (Ben‐Shlomo, 2017; Pérez‐Portela et al., 2012; Sheets, Cohen, Ruiz, & Rocha, 2016; Smith, 2012; Smith et al., 2015), but each researcher faced with multiple copies of the target sequence is forced to treat them in different ways due to limited analytical tools dealing with such intracolony variation. For example, Sheets et al. (2016) treated mitochondrial homoplasmy in *Botrylloides nigrum* (Herdman, 1886) individuals as two individuals, thereby artificially inflating the population size, whereas Smith (2012) highlighted the number of potential chimeras in *Didemnum vexillum* (Kott 2002), from New Zealand and Japan, but treated these as a separate experimental study due to the infancy of investigations of chimerism for this species. The main issue posed by chimerism for pathway identification is the potential for misinterpretation of genetic diversity and downstream population structure analyses.

Confounding the chimeric effect is the complex life history of colonial NIS. For instance, the life history of *D. vexillum* includes sexual and asexual reproductive strategies (Sakai et al., 2001), with seasonal regression in abundance and growth during winter months (Fletcher, Forrest, Atalah, et al., 2013; Valentine, Carman, Blackwood, & Heffron, 2007). The pelagic larval duration of most ascidians is limited to a few hours, reducing the potential for natural dispersal (Herborg, O'Hara, & Therriault, 2009; Morris & Carman, 2012; Osman & Whitlatch, 2007), but *D. vexillum* has been recorded as viable in the water column for up to 36 hr under laboratory conditions (Fletcher, Forrest, & Bell, 2013). Asexual reproduction by means of budding and fragmentation can also accelerate this species’ spread, as fragments may be less susceptible to competition and predation, and brooded larvae in fragments could be released prior to, or following, reattachment (Bullard et al., 2007). Furthermore, Morris and Carman (2012) found that under laboratory conditions, *D. vexillum* fragments can remain viable after suspension in the water column for three weeks, with only one zooid required for successful colony regrowth.

It is not surprising that we have limited power to resolve pathways of spread for NIS given the challenges posed by complex life histories, integrated with anthropogenic vector transport. In this study, we have used *D. vexillum* to investigate the effects of chimerism and seasonal regression on downstream population genetic analyses, to better understand their influence on interpretations and management decisions. As a dominant biofouler of artificial structures and due to its enhanced potential for spread (Fletcher, Forrest, & Bell, 2013), common chimeric formations (Smith, 2012), and winter regression, *D. vexillum* represents a valuable model for this study.

**Figure 1:**
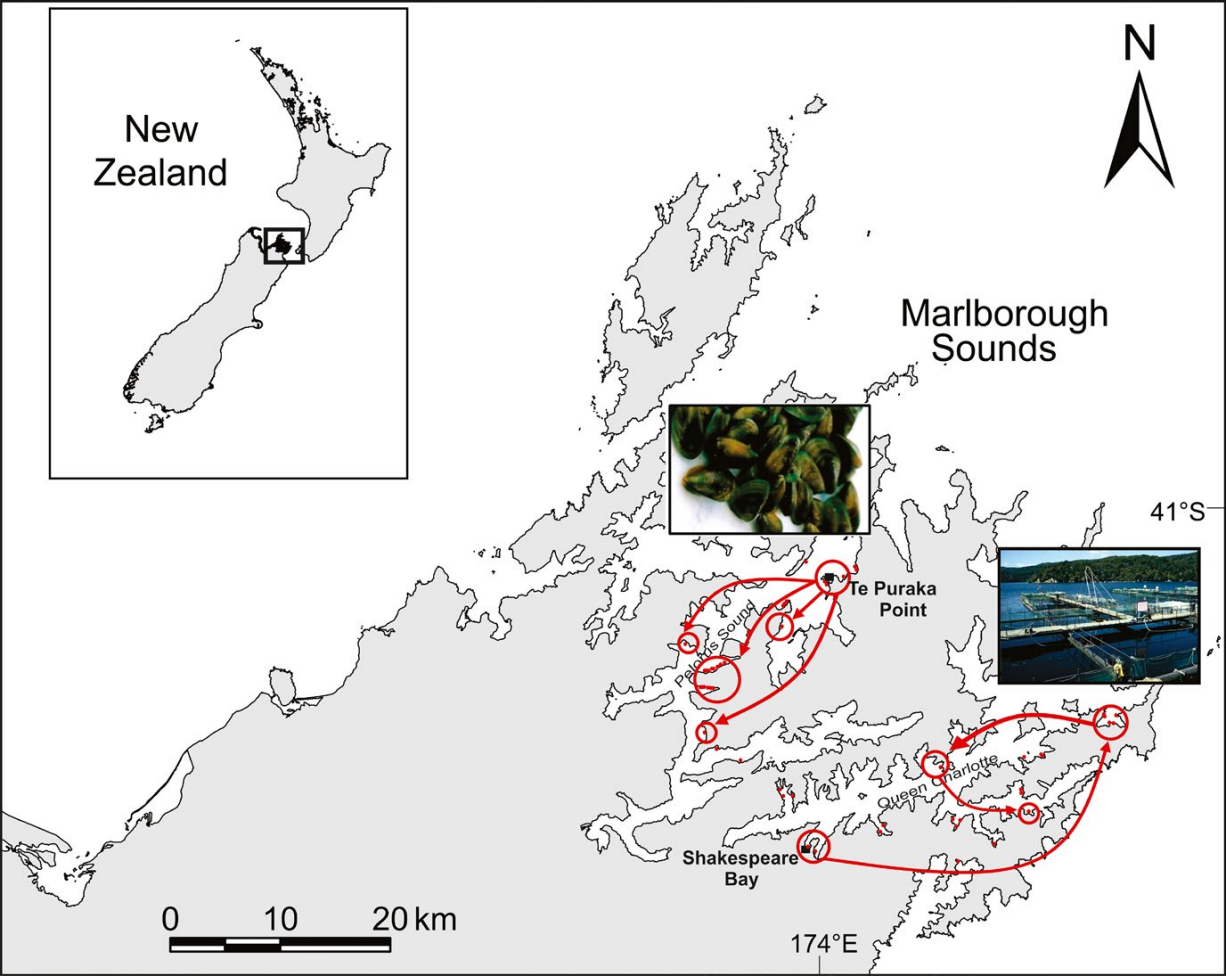
Map of the Marlborough Sounds, indicating the subsequent spread of *Didemnum vexillum* (red arrows) within Queen Charlotte Sound, following its initial incursion into Shakespeare Bay (black square), and within Pelorus Sound, following its incursion into Te Puraka Bay (black square). Spread was facilitated by the movement of aquaculture infrastructure and infected seed stock; images are located on the map in accordance with the areas influenced by these movements (map recreated, with photograph credit to Cawthron Institute, Lauren Fletcher). Unfilled red circles indicate the location of marine farms that were instrumental in the spread of *D. vexillum*

The New Zealand incursion of *D. vexillum* in the Marlborough Sounds region also provides a model system for this study. In New Zealand, *D. vexillum* has established in six major ports and an important aquaculture hub in the Marlborough Sounds region (Fletcher, Forrest, Atalah, et al., 2013; McDonald & Acosta, 2012) since its initial discovery in a North Island Harbour in 2001 (Coffey, 2001). Following its first recorded occurrence in the South Island in Shakespeare Bay, two events involving anthropogenic vectors were instrumental to the spread of *D. vexillum* around the Marlborough Sounds: the movement of an infected aquaculture structure to the outer regions of the Queen Charlotte Sound; and the regional transfer of infected mussel seed stock in the Pelorus Sound (Forrest & Hopkins, 2013; Figure 1). Subsequent local spread has been tentatively attributed to the movement of aquaculture equipment and vessels, as well as natural “stepping‐stone” dispersal between man‐made structures throughout the Sounds (Fletcher, Forrest, & Bell, 2013). However, this has not yet been investigated with genetic tools, providing an opportunity to test different scenarios of spread.

The primary goal of this region‐specific study was to investigate standard population genetic measures of an invader with a relatively well‐mapped pathway of invasion, but complex life history and genomic arrangement. Microsatellite markers were employed for fine‐scale spatial and temporal resolution. The four objectives were to: (a) investigate the genetic structure of *D. vexillum* colonies sampled in the Austral summer using a diploid dataset obtained from standard microsatellite scoring; (b) compare the observed genetic connectivity with predictions of dispersal generated from mathematical modeling; (c) assess the influence of chimerism on the genetic diversity and observed genetic connectivity; and (d) assess the influence of seasonal regression on the genetic structure of a subset of *D. vexillum* colonies.

## 2 MATERIALS AND METHODS

### 2.1 Sample collections

**Figure 2:**
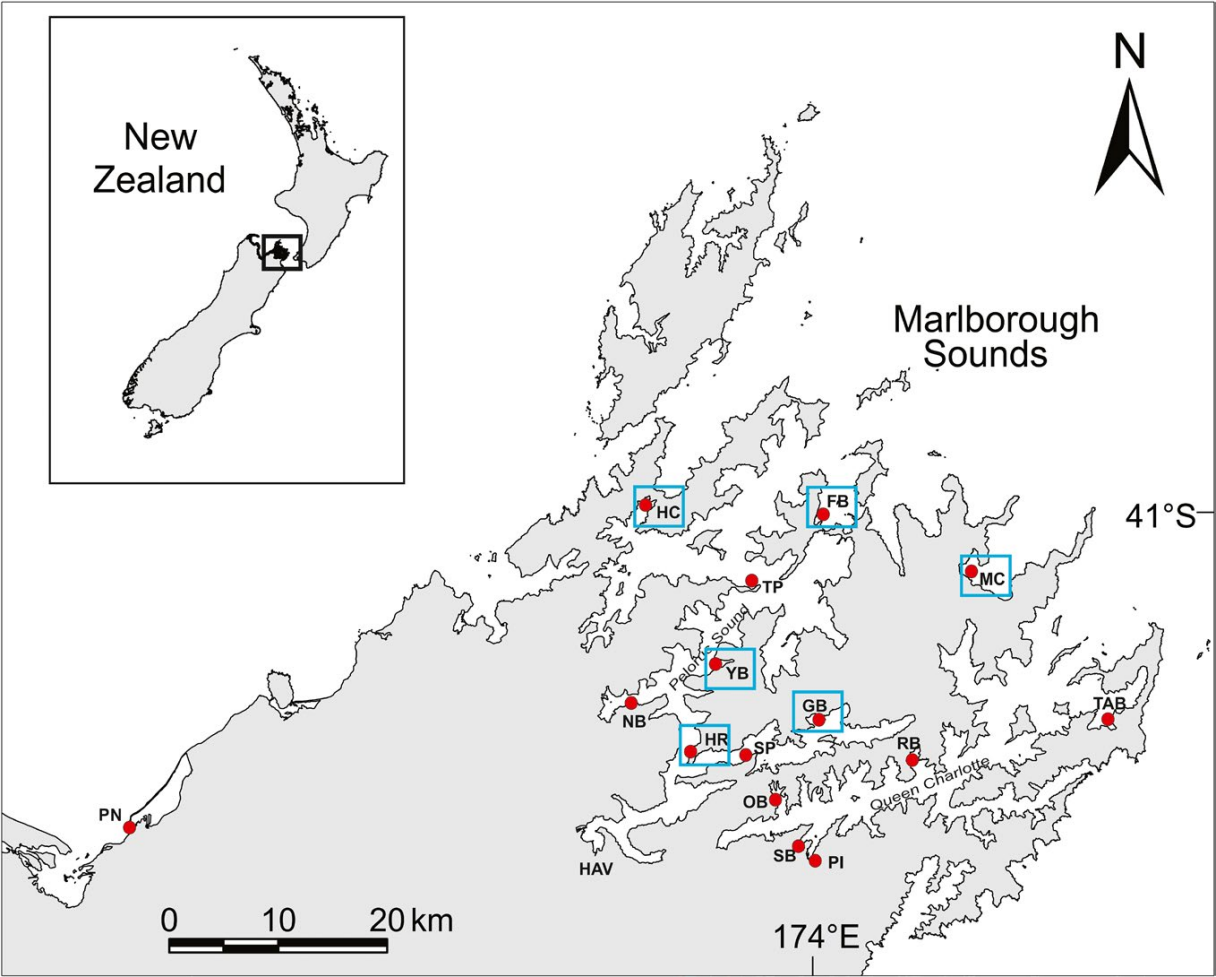
Map of the Tasman and Marlborough Sounds region where *Didemnum vexillum* samples were collected for microsatellite genotyping in the austral summer, April (red circles), and winter, August (red circles with blue outlined boxes). Each sampling site is labeled with initials. Queen Charlotte Sound sampling sites: SB = Shakespeare Bay, PI = Picton Marina, OB = Onahau Bay, RB = Ruakaka Bay, and TAB = Te Aroha Bay. Pelorus Sound (winter/summer) sampling sites: SP = Schnapper Point, GB = Goulter Bay, HR = Hikapu Reach, NB = Nydia Bay, YB = Yncyca Bay, TP = Tawero Point, HC = Hallam Cove, FB = Forsyth Bay, and MC = Melville Cove. Port Nelson sampling site: PN = Port Nelson

Tissue samples from colonies morphologically identified as *D. vexillum* were collected from artificial structures (mussel ropes, floats, wharfs) between January–March 2013 (austral summer, Figure 2) and early in August 2014 (austral winter, Figure 2). Thirty tissue samples were collected from structures at each site within a main aquaculture region and nearby port areas in the top of the South Island, New Zealand. Sampled sites in these areas included Port Nelson (*N* = 1 site, in summer), Pelorus Sound (*N* = 9 sites in summer and *N* = 6 sites in winter), and Queen Charlotte Sound (*N* = 5 sites in summer). The Port Nelson site represented a younger incursion, outside of the Marlborough Sounds region, sampled in summer to provide a more complete broad‐scale resolution for assignment tests. It was not used in the connectivity or seasonal comparisons. Samples were collected from colonies or colony fragments situated ≥2 m apart to minimize the chance of pseudoreplication due to sampling from clonally related colonies (Smith et al., 2012). Tissue samples (ca. 100–500 mg) were preserved in approx. 1.0 ml of 100% (v/v) ethanol and stored at −20°C.

### 2.2 DNA extraction and amplification

For each specimen, a 2‐mm^2^ section of tissue was cut for DNA extractions. The tissue was macerated using flame‐sterilized forceps and a scalpel. Total genomic DNA for polymerase chain reaction (PCR) amplification was extracted using a lithium chloride/chloroform extraction protocol (Gemmell & Akiyama, 1996). Extracted DNA samples were stored at −20°C.

### 2.3 Microsatellite processing

Thirteen polymorphic microsatellite loci were amplified in all samples. PCR amplifications were done using the Qiagen Type‐it Microsatellite PCR Kit and M13 tags to label each forward primer, with the addition of M13 fluorescent‐labeled primers (FAM, PET, VIC, NED). Loci were pooled for multiplexing (Schuelke, 2000) and assigned using the MULTIPLEX MANAGER 1.0 software (Holleley & Geerts, 2009) (Supporting information Table S1). The multiplexing protocol was performed with a final volume of 4 μl and contained 2x Type‐it Multiplex PCR Master Mix, 0.0216 µm of each M13‐labeled, locus‐specific forward primer, 0.0864 µm of each locus‐specific reverse primer, 0.135 µm of M13 5’‐end labeled with an Applied Biosystems (ABI) dye (FAM, NED, PET, or VIC), 0.82 μl of RNase‐free water, and 2 μl of 5.5–8.5 ng/µl diluted DNA.

The thermocycling parameters for all PCRs included an initial denaturation at 94°C for 15 min, 94°C for 30 s, 58°C for 90 s, and 72°C for 60 s for 8 cycles, followed by 89°C for 30 s, 56°C for 90 s, and 72°C for 60 s for 25 cycles, with a final 30‐min extension at 60°C (Schuelke, 2000). Post‐PCR products were diluted with 5 μl of Milli‐Q water, producing a total volume of 9 μl. A volume of 2 μl of the diluted PCR products was then taken from each multiplexed loci and pooled with other multiplexed loci to form genotyping groups (Supporting information Table S1), to a volume of 6–8 μl. To each genotype group, 10 μl formamide and 0.4 μl of GeneScan 500LIZ internal size standard ABI (per individual) were added for genotyping. Genotyping was resolved by the University of Canterbury Sequencing Service (Christchurch, New Zealand) on an ABI 3100 DNA analyzer. Alleles for each locus were scored using GENE MARKER v.1.6 (SoftGenetics LLC). Replicates (minimum 10% of the sample size) were assessed for amplification errors and the repeatability of scoring throughout the experimental process. One individual was used in every run as a positive control. Scores from individual amplifications were also compared to those acquired via multiplexing to detect possible multiplexing errors. To ensure genotyping accuracy, one negative and one positive control sample were included with each PCR run.

Given *D. vexillum* is able to form chimeric colonies, with the potential for more than two alleles per locus to be observed in a colony sample, the locus scores from the winter/summer samples were separated into two datasets. One dataset incorporated the two alleles with the highest peaks (diploid dataset), as per Smith (2012), and the other incorporated all detected peaks within 50% of the height of the two main peaks to produce the chimeric data.

### 2.4 Analyses

#### 2.4.1 Standard microsatellite summary

For the diploid dataset, all microsatellite markers and sampled populations (winter and summer) were assessed for deviations from Hardy–Weinberg equilibrium (HWE) and linkage disequilibrium (LD) using ARLEQUIN v.3.5.1.3 (Excoffier & Lischer, 2010). All calculations were conducted per locus and sampled population. For analyses conducted in ARLEQUIN v.3.5.1.3, 10,000 permutations were used and 95% confidence intervals for *F*‐statistics were obtained by bootstrapping over loci 20,000 times. In addition, MICRO‐CHECKER v.2.2.3 (Arif et al., 2010; Van Oosterhout, Hutchinson, Wills, & Shipley, 2004) was used to assess all loci for null alleles, as well as genotyping errors, such as large allele dropouts and stutter (1,000 randomizations). To account for multiple comparisons and control alpha inflation when two or more statistical tests on the same data were performed, a false discovery rate (FDR) correction was applied (Benjamini & Yekutieli, 2001; García, 2004; Narum, 2006). Using the following equation: $CPV=\alpha /\sum_{i=1}^{k}\left(1/i\right)$, the corrected *p*‐value (CPV) was obtained, where *α* = 0.05; *k* = the number of tests performed, and *i* = the *i*th observation (Narum, 2006). Values below this were considered significant for multiple comparisons.

##### Objective 1: Standard population genetic structure (summer diploid data)

To investigate the genetic structure among founder populations (early introductions into Queen Charlotte Sound) and secondary introductions (adjacent populations in Pelorus Sound), likelihood assignments were conducted in GenALEx v.6.5. Isolation by distance (IBD) among populations within Queen Charlotte Sound and Pelorus Sound was assessed using MANTEL tests in GenALEx v.6.5 (9,999 permutations) with a geographic Euclidean distance matrix (kilometers). The Port Nelson population was excluded from the IBD assessment, as spatial disharmony with sites would generate structural information, not pattern, in the results.

Estimates of allelic diversity incorporating allelic richness (*A_N_*), private allelic richness (*A*_PN_), and expected and observed heterozygosity (*H_E_*/*H_O_*) for loci and all populations were made using GenALEx v.6.5. The population‐specific inbreeding coefficient (*F*_IS_) was obtained using ARLEQUIN v.3.5.1.3. Rarefaction, based upon the lowest sample size (*N* = 19), was used to evaluate differences in allelic diversity (*A*_RN_, *A*_RP,_ and *H_E_*) among putative source and recipient sites (Pelorus Sound, Queen Charlotte Sound, Port Nelson), and among populations within sites, with single‐factor analysis of variance tests (ANOVAs) computed in R v.3.0.2 (R Core Team, 2013).

Genetic differentiation among Pelorus Sound, Queen Charlotte Sound, and Port Nelson was determined using a hierarchical analysis of molecular variance (AMOVA) performed in ARLEQUIN v.3.5.1.3. No more than two loci were missing per individual, and 10% missing data per loci were selected as an acceptable missing data rate (Pritchard, Stephens, & Donnelly, 2000).

##### Objective 2: Population connectivity assessed by genetic and modeled data (summer diploid data)

**Figure 3:**
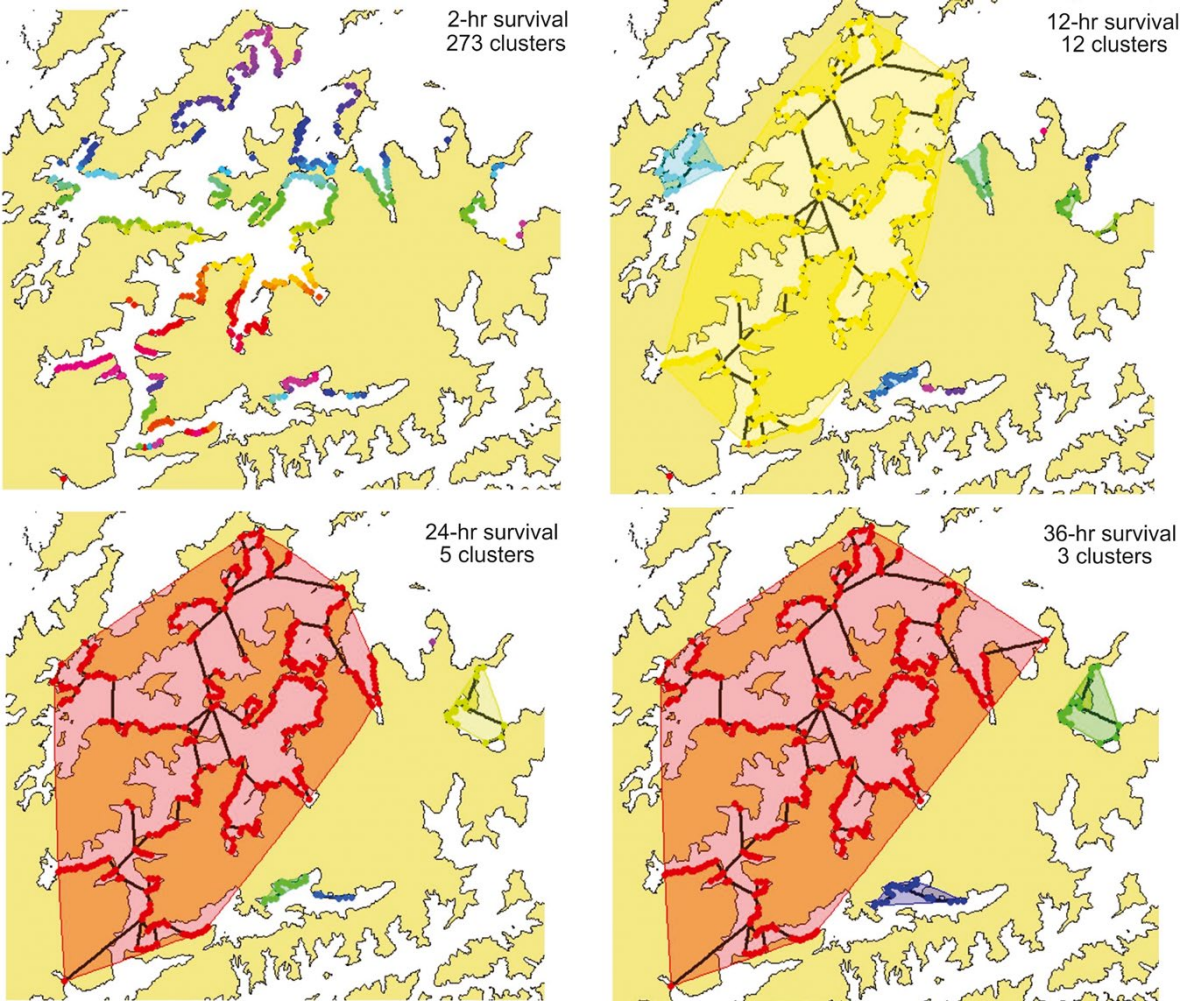
Maps of Pelorus Sound, displaying variable amounts of connectivity between mussel farms as generated by a connectivity matrix. Four different pelagic larval duration times and their associated clusters are presented (*a* = 2 hr, *b* = 12 hr, *c* = 24 hr, and *d* = 36 hr). Each cluster is represented by a different color, and connected clusters are denoted by lines and an overlaid, similar‐colored image

To generate the connectivity model, connectivity scores for artificial substrates throughout Pelorus Sound were obtained using a GIS‐based connectivity matrix. This matrix was constructed by incorporating estimated current velocities (Knight & Beamsley, 2012) and pelagic larval duration (PLD) (Fletcher, Forrest, Atalah, et al., 2013; Fletcher, Forrest, & Bell, 2013) using ArcMap 10.1 and R v.3.0.2. For this study, 2, 12, 24, and 36‐hr PLDs were selected to incorporate the entire range of *D. vexillum*'s natural dispersal capability (up to 36 hr, with 10% survival; Fletcher, Forrest, & Bell, 2013). Figure 3 provides a visualization of potential “stepping‐stone” movements between farms, with connectivity represented by lines and colors representing isolated “clusters”.

Locations sampled within Pelorus Sound were used to assess genetic differentiation between populations in relation to clusters generated from the mathematical connectivity model (testing 2, 12, 24, and 36‐hr PLD). Two of the PLD models (12‐ and 24‐hr) were tested with separate AMOVAs to assess genetic differentiation among sampled locations within and among resulting clusters. The clusters formed in the 24‐hr PLD were almost identical to the sampled farms within the 36‐hr PLD model (generated by the matrix), both models were represented by the 24‐hr model for further analysis. An AMOVA was not done on the 2‐hr PLD model as 273 clusters were formed, with each population sampled representing unique clusters.

To investigate differentiation between populations, pairwise *F*_ST_ (Wright, 1965) using ARLEQUIN v.3.5.1.3, and Jost's *D* (Jost, 2008) using the R package DEMEtics (Gerlach, Jueterbock, Kraemer, Deppermann, & Harmand, 2010), was calculated. Pairwise tests also provided a way to determine differentiation among populations within the 2‐hr PLD model. DEMEtics generated Jost's *D* by bootstrapping over loci 1,000 times; the package also applied a modified Benjamini–Hochberg false discovery rate correction for multiple statistical tests to P‐values for a family‐wise error of *α* = 0.05 (Benjamini & Yekutieli, 2001; Jueterbock, Kraemer, Gerlach, & Deppermann, 2011; Jueterbock, Kraemer, Gerlach, Deppermann, & Jueterbock, 2013; Narum, 2006). A Pearson correlation between FST and Jost's *D* values was used to ensure both estimators provided comparable information.

##### Objective 3: Chimeric comparison of standard population genetic structure and connectivity (summer chimera dataset)

To assess the influence of chimerism, the raw allele data were rescored so that additional allele peaks detected by the ABI 3100 DNA analyzer within 50% of the height of the two main allele peaks (the diploid dataset) were incorporated into one dataset. These were then treated as polyploid data as it was not possible to isolate the different individuals or genotypes in chimeric results. POLYSAT in R v.3.0.2 (Clark & Jasieniuk, 2011) was then used to determine differences in the allelic richness and diversity of *D. vexillum* colonies and PLD models when incorporating polyploid data compared to excluding it. In the POLYSAT analysis, all individuals were assumed to be tetraploid. Ploidy was set to four, and missing values were given a set value. POLYSAT was also used to generate pairwise FST comparisons for the winter and summer polyploid data. A single‐factor analysis of variance test (ANOVAs) computed in R v.3.0.2 was used to test the significance of any differences.

To visualize the impact of incorporating chimeric data, population distance matrices were used to generate separate principal coordinates analyses (PCoA) in GENALEX v.6.5 for both diploid and polyploid datasets derived from the summer sampling period.

##### Objective 4: Seasonal comparison of standard population genetic structure and connectivity (winter diploid and chimeric datasets)

A subset of the *D. vexillum* sites sampled in Pelorus Sound during summer were resampled in winter (August 2014) to assess the role of dieback on genetic diversity and patterns of genetic population connectivity (Figure 2). The populations chosen for resampling were based on industry boat movements and the presence of Didemnum. All genetic analyses, except for isolation by distance, were repeated for the winter diploid dataset. A winter polyploid dataset was created, and the influence of chimeric data was analyzed as previously described. Genetic differentiation between seasons was determined using a hierarchical analysis of molecular variance (AMOVA) performed in ARLEQUIN. No more than two loci were missing per individual, and 10% missing data per loci were selected as an acceptable missing data rate (Pritchard et al., 2000).

## 3 RESULTS

### 3.1 Standard microsatellite summary

Eight of the thirteen polymorphic microsatellite loci amplified consistently across all sampled locations (for summer and winter samples), with 3 (Dvex01) to 13 (Dvex30 and Dvex33) alleles observed per locus (Supporting information Table S1). Allele scoring was repeatable at 95% for the positive control across all loci. Chimeric colonies were identified across all populations (ranging from 17% to 48%; Table 1).

**Table 1:** Estimates of genetic diversity for fifteen *Didemnum vexillum* populations sampled in summer across three New Zealand sites (Pelorus Sound, Queen Charlotte Sound, and Port Nelson) and six populations subsampled in winter from Pelorus Sound (bold faces denote summer areas which were subsampled winter populations) using all loci

Season	Site	*N*	A_N_ (dip)	A_N_ (poly)	*A*_RN_	*A*_RP_	*H_O_*	*H_E_*	*F*_IS_	CC
Summer	*Pelorus Sound*	*240*	*4.93 (±0.08)*	*5.32 (±0.14)*	*3.88 (±0.04)*	*0.15 (±0.02)*	*0.66 (±0.01)*	*0.57 (±0.00)*	*−0.20824 ns*	82 (24%)
	**Goulter**	**26**	**5.33 (±0.80)**	**5.54 (±0.60)**	**4.25 (±0.61)**	**0.12 (±0.08)**	**0.65 (±0.08)**	**0.61 (±0.06)**	**−0.05656 ns**	**11 (42%)**
	**Hikapu**	**29**	**4.50 (±0.76)**	**4.75 (±0.59)**	**3.76 (±0.63)**	**0.01 (±0.01)**	**0.68 (±0.09)**	**0.55 (±0.07)**	**−0.27015 ns**	**7 (24%)**
	**Yncyca**	**26**	**4.50 (±0.76)**	**5.00 (±0.65)**	**3.51 (±0.54)**	**0.16 (±0.12)**	**0.57 (±0.12)**	**0.52 (±0.09)**	**−0.15516 ns**	**5 (19%)**
	**Hallam**	**21**	**4.17 (±0.60)**	**4.88 (±0.67)**	**3.58 (±0.45)**	**0.00 (±0.00)**	**0.68 (±0.10)**	**0.54 (±0.05)**	**−0.35622 ns**	**10 (47%)**
	**Forsyth**	**28**	**4.83 (±0.65)**	**5.62 (±0.73)**	**3.63 (±0.49)**	**0.13 (±0.10)**	**0.66 (±0.09)**	**0.55 (±0.06)**	**−0.22754 ns**	**9 (32%)**
	**Melville**	**29**	**6.33 (±1.36)**	**6.14 (±1.00)**	**4.50 (±0.81)**	**0.45 (±0.15)**	**0.67 (±0.06)**	**0.64 (±0.06)**	**−0.06586 ns**	**12 (41%)**
	Schnapper	24	5.16 (±0.80)	5.50 (±0.63)	4.27 (±0.64)	0.22 (±0.14)	0.71 (±0.08)	0.60 (±0.06)	−0.24687 ns	6 (25%)
	Nydia	30	5.00 (±0.85)	5.37 (±0.80)	4.03 (±0.58)	0.16 (±0.01)	0.68 (±0.09)	0.58 (±0.06)	−0.19867 ns	11 (37%)
	Tawero	27	4.50 (±0.62)	5.25 (±0.67)	3.44 (±0.54)	0.09 (±0.06)	0.72 (±0.06)	0.56 (±0.04)	−0.29716 ns	11 (40%)
	*Queen Charlotte*	*124*	*4.53 (±0.03)*	*5.00 (±0.14)*	*3.74 (±0.05)*	*0.11 (±0.01)*	*0.56 (±0.00)*	*0.63 (±0.01)*	*−0.18075 ns*	44 (35%)
	Picton	24	4.50 (±0.76)	5.14 (±0.54)	3.69 (±0.63)	0.08 (±0.07)	0.62 (±0.07)	0.57 (±0.05)	−0.15989 ns	5 (20%)
	Shakespeare	26	5.00 (±0.82)	5.25 (±0.62)	4.02 (±0.61)	0.19 (±0.10)	0.68 (±0.08)	0.61 (±0.04)	−0.13537 ns	12 (46%)
	Ruakaka	28	4.50 (±0.67)	4.88 (±0.58)	3.45 (±0.38)	0.07 (±0.07)	0.64 (±0.11)	0.52 (±0.06)	−0.26600 ns	11 (39%)
	Te Aroha	27	4.50 (±0.67)	5.25 (±0.67)	3.66 (±0.41)	0.07 (±0.05)	0.61 (±0.06)	0.56 (±0.04)	−0.13392 ns	7 (26%)
	Onahau	19	4.17 (±0.65)	4.50 (±0.53)	3.88 (±0.59)	0.11 (±0.10)	0.62 (±0.10)	0.55 (±0.06)	−0.20855 ns	9 (47%)
	*Port Nelson*	24	5.00 (±0.53)	5.13 (±0.58)	4.00 (±0.40)	0.17 (±0.17)	0.73 (±0.05)	0.62 (±0.03)	−0.31129 ns	9 (36%)
	Overall	364	4.78 (±0.14)	5.09 (±0.15)	3.83 (±0.08)	0.13 (±0.03)	0.65 (±0.01)	0.57 (±0.01)	−0.19842 ns	135 (35%)
*Winter*	*Pelorus Sound*	*146*	*4.44 (±0.26)*	*4.84 (±1.71)*	*3.77 (±0.19)*	*0.22 (±0.07)*	*0.60 (±0.05)*	*0.56 (±0.03)*	*−0.16541 ns*	54 (32%)
	Goulter	27	4.75 (±0.53)	5.13 (±1.81)	3.89 (±1.37)	0.21 (±0.07)	0.67 (±0.07)	0.61 (±0.05)	−0.13537 ns	9 (33%)
	Hikapu	22	4.25 (±0.53)	4.38 (±1.55)	3.69 (±1.0)	0.19 (±0.07)	0.56 (±0.07)	0.51 (±0.06)	−0.18216 ns	5 (17%)
	Yncyca	22	3.63 (±0.50)	3.75 (±1.33)	3.19 (±1.13)	0.06 (±0.02)	0.47 (±0.10)	0.49 (±0.08)	−0.10407 ns	12 (48%)
	Hallam Cove	25	5.25 (±0.62)	6.00 (±2.12)	4.49 (±1.59)	0.50 (±0.18)	0.76 (±0.05)	0.66 (±0.03)	−0.23636 ns	13 (48%)
	Forsyth	28	4.88 (±0.40)	5.63 (±1.99)	3.96 (±1.40)	0.29 (±1.10)	0.69 (±0.08)	0.59 (±0.03)	−0.29375 ns	10 (32%)
	Melville	22	3.88 (±0.40)	4.13 (±1.46)	3.37 (±1.19)	0.05 (±0.02)	0.46 (±0.10)	0.52 (±0.05)	−0.04076 ns	5 (17%)

Notes

Statistics shown for summer and winter are as follows: *N*, sample size; *A_N_* (dip), mean number of alleles from the diploid dataset; *A_N_* (poly), mean number of alleles from the polyploid dataset; A_RN,_ rarefied mean allelic richness; *A*_RP,_ rarefied mean private allelic richness based on sample sizes of *N* = 19; *H_O_*_,_ observed heterozygosity; *H_E_*_,_ expected heterozygosity under Hardy–Weinberg equilibrium; *F*_IS,_ the inbreeding coefficient; and CC, the proportion of chimeric colonies detected in populations. All means ± *SE*.

N.B: ns: FIS not significant at *p* < 0.05.

All populations, across seasons, showed significant deviations from Hardy–Weinberg equilibrium (HWE) for some of the loci; this was primarily due to homozygote deficits. MICRO‐CHECKER suggested no evidence of stutter or genotyping errors across all sampled populations, but detected the potential presence of null alleles for four markers, though this was not consistent across all populations within each locus**.** To limit the influence of the detected deviations from HWE and suggested null alleles, subsequent analyses were repeated with and without loci Dvex10 and Dvex19, these two loci were selected from the four as they showed the highest presence of null alleles, evident in ≥half of all the populations. Overall, genetic patterns were consistent when loci Dvex10 and Dvex19 were included and removed (Watts, 2014, *unpublished*). Therefore, the full set of loci was used for all final analyses.

#### 3.1.1 Objective 1: Standard population genetic structure (summer diploid data)

Of the of 420 linkage disequilibrium tests, 11 (2.6%) showed evidence for loci pairs with linkage disequilibrium after FDR correction (CPV = 0.008). The test for linkage disequilibrium in ARLEQUIN assumes Hardy–Weinberg proportions of genotypes and therefore the significance of these tests may be a result of departures from HWE (Excoffier & Slatkin, 1998). However, loci pairs were not consistently linked across populations, and therefore physical chromosomal linkage appears unlikely, and all markers were considered as independent replicates of the *D. vexillum* genome.

There was a total range of 4 (Dvex01) to 13 (Dvex30 and Dvex33) alleles for the eight polymorphic loci (Supporting information Table S1, *A*_N_, summer). Overall, genetic diversity for the three primary locations sampled (Pelorus Sound, Queen Charlotte Sound, and Port Nelson) was consistently high, with average observed and expected heterozygosity ranging from 0.57 ± 0.12 to 0.73 ± 0.05 and 0.52 ± 0.09 to 0.64 ± 0.05, respectively (Table 1, *H_O_* and *H_E_*, summer). Queen Charlotte Sound populations exhibited the lowest levels of observed and expected heterozygosity on average (*H_O_* = 0.56 ± 0.00, *H_E_* = 0.63 ± 0.01, Table 1, summer). While *t* tests showed no significant differences between observed and expected heterozygosity, all populations exhibited homozygote deficiency within MICRO‐CHECKER analyses, and there was no evidence of significant inbreeding (Table 1, summer). It should be noted that null alleles can cause a heterozygote to be mistakenly read as homozygote, that is, can result in homozygote excess, which is not observed in these data. Allelic and private allelic richness ranged from 3.45 ± 0.38 (Ruakaka Bay) to 4.50 ± 0.81 (Melville Cove) and from 0.00 ± 0.00 (Hallam cove) to 0.45 ± 0.15 (Melville Cove), respectively (Table 1, summer). On average, allelic richness and private richness were similar across all sites, although the Port Nelson site had a slightly higher proportion of private alleles (0.17 ± 0.17, Table 1, summer). ANOVA analyses showed no significant differences (*p* > 0.05) in allelic diversity (*A*_RN_, *A*_RP,_ and *H_E_*), when compared across sites (Pelorus Sound, Queen Charlotte Sound, and Port Nelson) and across populations within Pelorus Sound and Queen Charlotte Sound.

There was no evidence for isolation by distance among summer populations in Pelorus Sound and Queen Charlotte Sound (rxy = 0.132, *p* = 0.260). Similarly, there was no evidence or trend for isolation by distance among the Pelorus Sound populations and among Queen Charlotte Sound populations.

AMOVA results revealed significant genetic differentiation among sites (Pelorus Sound, Queen Charlotte Sound, and Port Nelson) and among populations within sites, although this explained only a small proportion of the genetic variation (1.48% and 3.49%, respectively, Table 2, summer). Significant variation within populations was detected (*F*_ST_ = 0.04975; *p < *0.001; Table 2, summer results), and this explained a large proportion of the variation in the data (95%, Table 2, summer results). However, for all levels of the AMOVA, F_ST_ values were low (*F*_ST_ = 0.05), indicating little to moderate genetic differentiation, as defined by Wright (1978). When the Port Nelson population was removed from AMOVA analyses, no significant genetic variation was detected among Queen Charlotte Sound and Pelorus Sound populations (Table 2, summer).

**Table 2:** Analysis of molecular variance (AMOVA) results for *Didemnum vexillum* summer and winter microsatellite data for all loci, including *F*‐statistics (*F*_CT_, *F*_SC_, and *F*_ST_), associated 95% confidence intervals, and percentages of explained variation

	*F*_CT_	*F*_SC_	*F*_ST_
Summer results			
Queen Charlotte Sound, Pelorus Sound, and Port Nelson			
*F*‐statistic	0.0148*	0.03547***	0.04975***
(95% CI)	(0.00439, 0.02943)	(0.02585, 0.05544)	(0.03252, 0.07588)
Variation (%)	1.48	3.49	95.03
Queen Charlotte Sound and Pelorus Sound			
*F*‐statistic	0.0108 ns	0.03515***	0.0456***
(95% CI)	(0.00321, 0.01881)	(0.02110, 0.05578)	(0.02871,0.06949)
Variation (%)	1.09	3.48	95.44
Pelorus Sound only—24‐hr cluster			
*F*‐statistic	−0.00791 ns	0.04009***	0.03250***
(95% CI)	(−0.02419, 0.00475)	(0.02205, 0.06593)	(0.01921, 0.05224)
Variation (%)	−0.79	4.04	96.75
Pelorus Sound only—12‐hr cluster			
*F*‐statistic	−0.01669 ns	0.04575***	0.02982***
(95% CI)	(−0.02676, 0.0098)	(0.02856, 0.07010)	(0.01901, 0.05108)
Variation (%)	−1.67	4.65	97.02
Winter results			
Pelorus Sound—Summer versus Winter			
*F*‐statistic	0.028***	0.55***	0.0819***
(95% CI)	(0.06096, 0.060969)	(0.02376, 0.07078)	(0.12231, 0.12231)
Variation (%)	2.81	5.54	91.8
Pelorus Sound only—24‐hr cluster			
*F*‐statistic	−0.0088***	0.07305***	0.06489 ns
(95% CI)	(−0.05130, 0.01120)	(0.02443, 0.09978)	(0.02823, 0.08344)
Variation (%)	−0.88017	7.36924	93.51093
Pelorus Sound only—12‐hr cluster			
*F*‐statistic	−0.02741 ns	0.08874***	0.06376***
(95% CI)	(−0.09690, 0.00680)	(0.02011, 0.13197)	(0.02952, 0.08014)
Variation (%)	−2.74	9.12	93.62

Four separate AMOVA results, looking at differences across sites (Pelorus Sound, Queen Charlotte Sound, and Port Nelson) and across two different cluster groupings (24‐hr and 12‐hr pelagic larval duration times).

ns: not significant.

^*^ *p* < 0.05, ^**^ *p* < 0.01, ^***^ *p* < 0.001.

**Figure 4:**
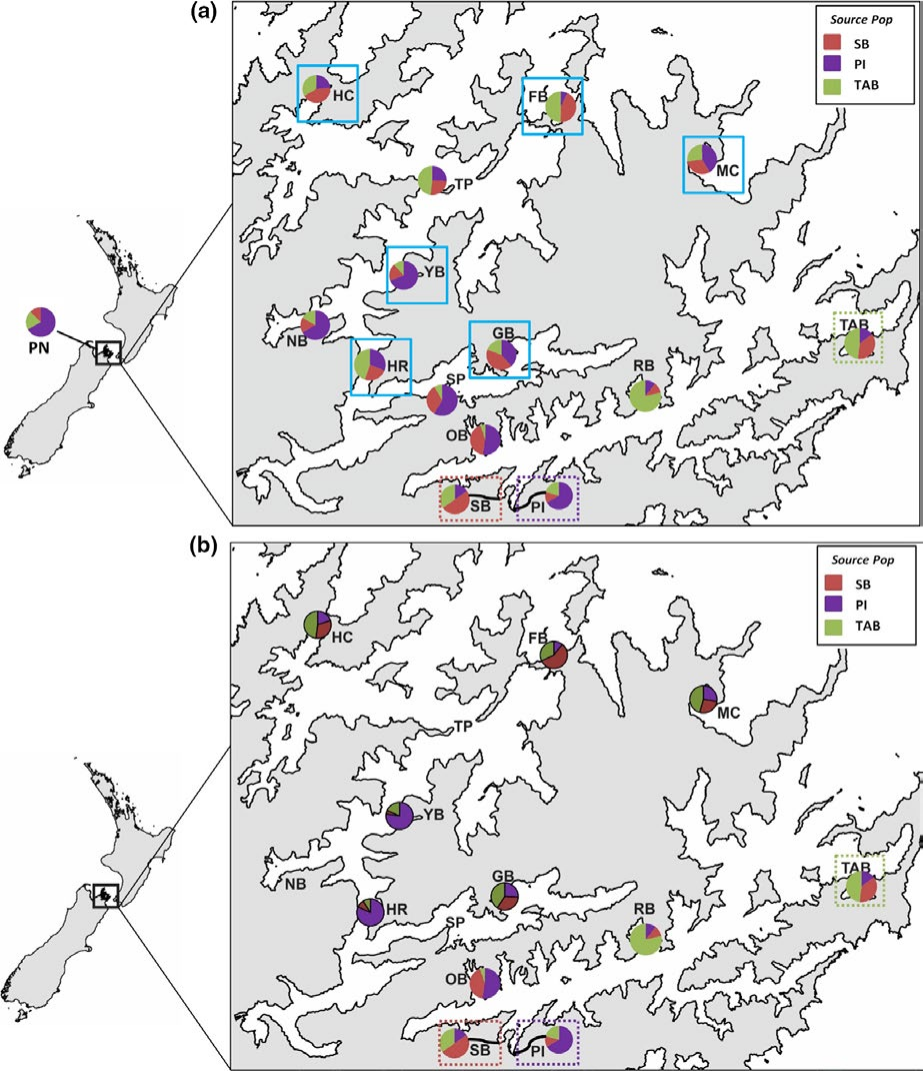
Results from likelihood assignment tests for 15 summer *Didemnum vexillum* populations taken from Pelorus Sound, Queen Charlotte Sound, and Port Nelson (a), and a subsample of six winter samples taken from Pelorus Sound (a, winter sample sites outlined by blue boxes in a and b, winter sites identified by bold outlined pie charts and slightly darker shading). Results display the proportion of populations within Pelorus Sound, Queen Charlotte Sound, and Port Nelson assigned to potential source populations located within Queen Charlotte Sound. The three source populations sampled and tested were as follows: SB = Shakespeare Bay, represented by red proportions of the pie charts; PI = Picton Marina, represented by purple proportions of the pie charts; and TAB = Te Aroha Bay, represented by the green proportion of the pie charts

Likelihood assignments, where the three founder populations were preset and Pelorus Sound samples were assigned to these, indicated patchy substructure and high levels of admixture among all populations. Consequently, it was not possible to definitively identify a single founder population for Pelorus Sound (Figure 4a). However, all three founder populations had ≥50% “self” assignment (Figure 4a). In addition, there was evidence of clustering in the assignment, with the outer sound populations predominantly assigned to Te Aroha Bay, and the inner sound populations, as well as the Port of Nelson population predominantly assigned to Picton (Figure 4a).

#### 3.1.2 Objective 2: Population connectivity assessed by genetic and modeled data (summer diploid data)

AMOVA results revealed no significant genetic differentiation among clusters for the 24‐ and 12‐hr PLD models (24‐hr *F*_CT_ = −0.00791, 12‐hr *F*_CT_ = −0.01669, *p > *0.05; Table 2, summer). However, significant genetic differentiation was found among populations within clusters for the 24‐hr and 12‐hr PLD models (24‐hr *F*_SC_ = 0.04009, 12‐hr *F*_SC_ = 0.04575, *p* < 0.001; Table 2, summer). Most of the variation (%) within the data was found within populations (97%; Table 2, summer). A lack of genetic differentiation among clusters was mirrored in the pairwise fixation index (*F*_ST_) and distance measures (Jost's *D*), which showed that each cluster group (e.g., Clust 1) had at least one population present in another cluster group (e.g., Clust 2), from which it was not significantly differentiated (Supporting information Table S2a–c). Pearson correlation between *F*_ST_ and Jost's *D* was highly significant (*r* = 0.970, *p* < 0.01), ensuring both estimators provided comparable results. Genetic differentiation was therefore not reflective of PLD and current movements alone.

#### 3.1.3 Objective 3: Chimeric comparison of standard population genetic structure and connectivity (summer chimera dataset)

All populations exhibited chimerism at 17%–48% (Table 1). While technical artifacts can sometimes be created by programs such as POLYSAT, the allelic diversity for the chimeric dataset (polyploid data) was not significantly different from the diploid dataset (*t* = 2.15, *p* = 0.803), providing some confidence in the program. However, for chimeric colonies, the frequency of more common alleles was reduced, less common allele frequencies were increased, and rare alleles were introduced (Supporting information Table S3 and S4).

**Figure 5:**
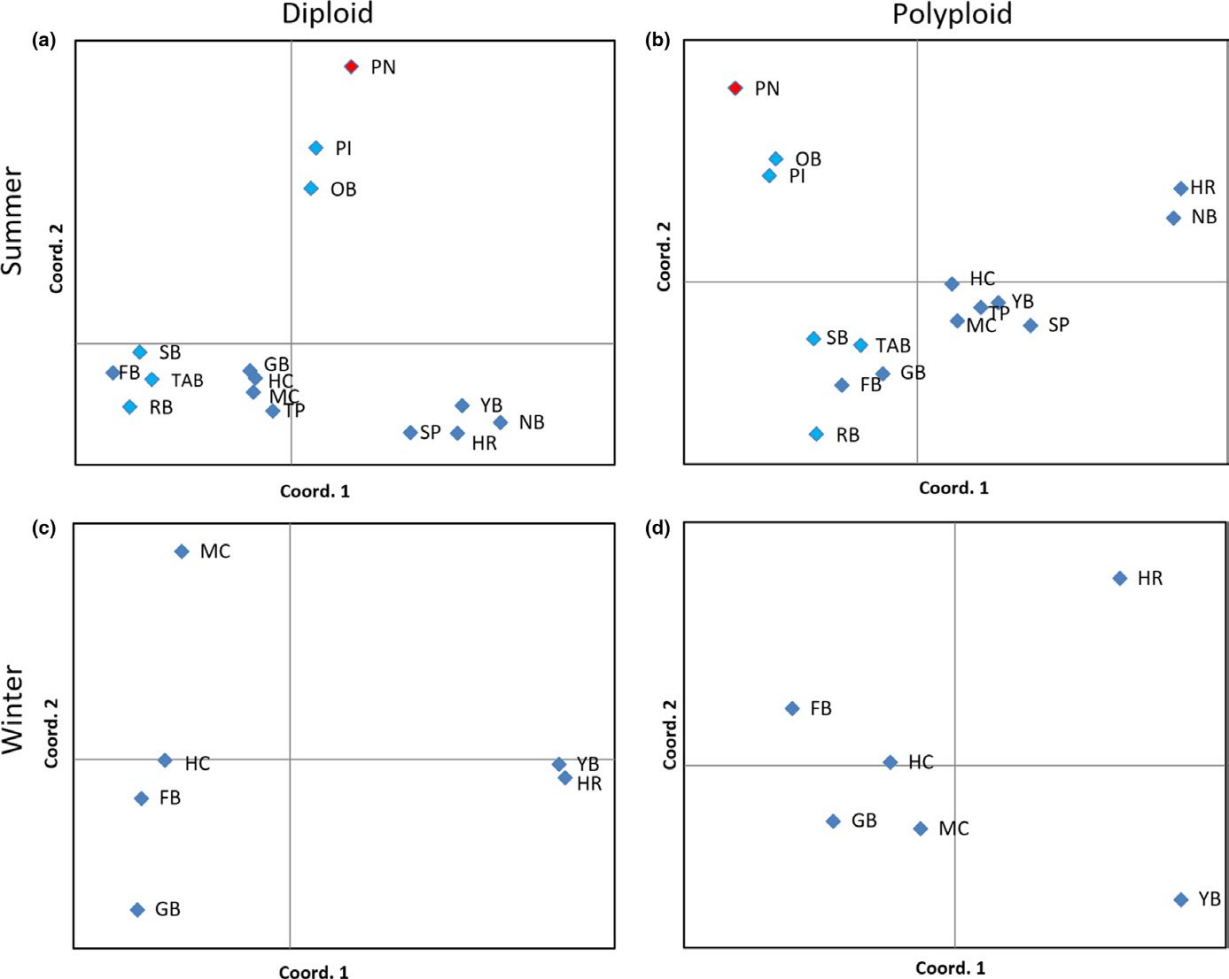
Separate principal coordinates analyses (PCoA) for summer diploid (a) and polyploid (b) datasets, and winter diploid (c) and polyploid (d) datasets. Each sampling site is labeled with initials. Queen Charlotte Sound sampling sites: SB = Shakespeare Bay, PI = Picton Marina, OB = Onahau Bay, RB = Ruakaka Bay, and TAB = Te Aroha Bay (light blue color). Pelorus Sound (winter/summer) sampling sites: SP = Schnapper Point, GB = Goulter Bay, HR = Hikapu Reach, NB = Nydia Bay, YB = Yncyca Bay, TP = Tawero Bay, HC = Hallam Cove, FB = Forsyth Bay, and MC = Melville Cove (dark blue color). Port Nelson sampling site: PN = Port Nelson (red color). Population distance matrices were used to generate PCoA plots

Results generated from the summer diploid PCoA analysis showed five separate “groupings.” The Port Nelson population appeared as an outlier from other sampled populations (Figure 5a). Two Queen Charlotte Sound sites (Picton and Onahau) were evidently more similar to one another than to all other populations, and the three remaining groups showed a mix of Queen Charlotte Sound and Pelorus Sound populations (Figure 5a). In contrast, the summer polyploid PCoA analyses showed slightly different “groupings” (Figure 5b). The Port Nelson population remained an outlier, as did Onahau and Picton Bays. However, Yncyca and Schnapper Point sites became mixed with the main grouping (Figure 5b), where they were previously clustered with Hikapu Reach and Nydia Bay (Figure 5a).

#### 3.1.4 Objective 4: Seasonal comparison of standard population genetic structure and connectivity (winter diploid and chimeric datasets)

##### Winter diploid

For the winter samples, there was a total range of 3 (Dvex01) to 9 (Dvex03) alleles for the eight polymorphic loci (Supporting information Table S1, A_N_, winter). Of the 168 linkage disequilibrium tests completed for the winter diploid dataset, four (2.4%) showed evidence for loci pairs with linkage disequilibrium after FDR correction (CPV = 0.009), spread across populations sampled in Pelorus Sound. Overall, genetic diversity was consistently higher in winter than summer, with a wider range of average observed and expected heterozygosity and a slightly higher observed heterozygosity, ranging from 0.46 (±0.10) to 0.76 (±0.05) and 0.49 (±0.08) to 0.66 (±0.03), respectively (Table 1, winter). The ranking of heterozygosity measures in the populations sampled in winter remained mostly consistent with the summer samples (Supporting information Table S1, summer and winter), and *t* tests showed no significant differences between observed (*t* = 1.81, *p* = 0.19) and expected heterozygosity (*t* = 1.81, *p* = 0.44) for winter and summer populations. All winter populations exhibited homozygote deficiency, with no evidence of significant inbreeding (Table 1, winter). The range of allelic and private richness was similar across seasons, although winter samples were slightly lower for allelic richness and higher for private allelic richness, ranging from 3.19 ± 1.13 (Yncyca) to 3.96 ± 1.4 (Forsyth) and 0.05 ± 0.02 (Melville) to 0.50 ± 0.18 (Hallam Cove) respectively (Table 1, winter).

Separate ANOVA tests showed no significant differences in allelic diversity (A_RN_, A_RP,_ and H_E_) within winter sites or between summer and winter sites.

AMOVA results revealed significant genetic differentiation between the summer and winter populations (*F*_CT_ = 2.81, *p* < 0.001), and among populations within sites (*F*_SC_ = 0.55, *p* < 0.001), although this only explained a small proportion of the genetic variation (Table 2, winter results, 2.81% (*F*_CT_) and 5.54% (*F*_SC_), respectively). Significant variation was also detected within the summer and winter groups, which explained 91.8% of the variation in the data (Table 2, winter results). For all levels of the AMOVA, *F*_ST_ values were low (*F*_ST_ ≤ 0.08), indicating little to moderate genetic differentiation.

Likelihood assignment patterns for the winter populations were different from the summer patterns, as dominant assignment to the three “preset” founder populations varied for all six populations sampled (Figure 4b). The clustering of assignments dissolved, and in two cases (Yncyca Bay and Hikapu Reach), one of the three preset populations was almost entirely lost from the sample.

AMOVA winter results for the connectivity model varied from those in summer and were better aligned with the model results, showing significant genetic differentiation among clusters for the 24‐hr PLD model (*F*_CT_ = −0.0088, *p* < 0.001; Table 2, winter results). There was also significant genetic differentiation among populations within clusters for the 24‐hr PLD model (*F*_SC_ = 0.07305, *p* < 0.001; Table 2). While 94% of the variation was found within clusters (Table 2), genetic differentiation was not significant (*F*_ST_ = 0.06489, *p* > 0.05; Table 2). Similarly, significant genetic differentiation was not detected among clusters for the 12‐hr PLD models (*F*_CT_ = −0.02741, *p* > 0.05; Table 2).

Genetic differentiation detected among clusters from AMOVA results was mirrored in the pairwise fixation index (*F*_ST_) and distance measures (Jost's *D*) (Supporting information Table S5a, b, and c), and was similarly reflective of the PLD and current movement represented by the connectivity model.

##### Winter chimeric data

The proportion of chimeric colonies identified in winter (17%–48%) was similar to summer (19%–47%). Overall, no significant differentiation was found between summer and winter populations when incorporating chimeric data (*F*_ST_ = 0.02, *p* > 0.05), and significant differences in the overall allele diversity and frequency were also not found.

Results generated from the winter diploid PCoA analysis showed four separate “groups” (Figure 5c). Melville Cove appeared as an outlier with less similarity to all other sampled populations, as did Goulter Bay (Figure 5c). Hallam Cove and Forsyth were grouped together, as were Yncyca and Hikapu Reach, each appearing more similar to one another than all other populations (Figure 5c). In contrast, the chimeric data changed these groupings, all samples appeared different to each other, with no obvious “groups” appearing in the winter polyploid PCoA analysis (Figure 5d).

## 4 DISCUSSION

In this study, we have assessed the influence of chimerism and winter dieback on the interpretation of standard population genetic structure and connectivity analyses of the nonindigenous colonial ascidian *Didemnum vexillum*. Although overall allelic changes were not significant with the inclusion of chimerism and winter dieback, the changed distribution of alleles did alter downstream population analyses and interpretation for both sets of data.

The allelic richness and the observed and expected heterozygosity estimates from the summer diploid samples were consistent with microsatellite studies for *D. vexillum* and other colonial ascidians, such as *Botrylloides violaceus,* and *Botryllus schlosseri*, where chimeras were excluded from the data (Abbott, Ebert, Tabata, & Therriault, 2011; Ben‐Shlomo, Douek, & Rinkevich, 2001; Bock, Zhan, Lejeusne, MacIsaac, & Cristescu, 2011). An excess of heterozygosity has also been detected in solitary self‐fertilizing ascidians *Corella inflata* and *Chelyosoma productum,* sampled in Washington (Cohen, 1990). In contrast to our study, deviations from HWE were not detected within *D. vexillum* populations from samples taken across New Zealand. Those results were pooled from across New Zealand to present one “area,” so it is plausible that the scale of sampling reduced their ability of to detect heterozygote excess or deficiency present within localized populations.

The genetic structure among populations within the Marlborough Sounds was lower than observed among *D. vexillum* populations from the east and west coasts of America (Hess, Swalla, & Moran, 2009), as would be expected from geographic parameters and colonization history of the two studies. In 2012, Smith *et al*. sequenced the COI gene for a single New Zealand and Japanese population of *D. vexillum*. The authors concluded that the New Zealand population suffered reduced haplotype diversity from the colonization process and exhibited increased potential for fusion. Eighty percent of the experimental colonies from New Zealand exhibited fusion compared to only 27% of colonies tested in Japan. This high fusion potential of the New Zealand colonies suggests 17%–48% chimerism observed in our data was within an expected range.

To our knowledge, only one other study has partially incorporated chimerism into downstream analyses, and this was for the colonial ascidian *Botrylloides nigra* (Sheets et al., 2016). Homoplasy observed in *B. nigra* samples was incorporated by treating homoplasmic individuals as two separate individuals. This inclusion added three unique haplotypes to the data, which were spread between two oceans. When the chimeric individuals were removed from diversity analyses, two populations greatly decreased in diversity. The downstream analyses were not conducted with and without the chimeras so the influence on statistical interpretations was not assessed (Sheets et al., 2016).

In our study, the inclusion of chimeras also altered the distribution of allelic diversity, such that the frequency of more common alleles in some populations was reduced, less common allele frequencies were increased, and rare alleles were introduced. The introduction of rare alleles (frequencies <0.01) is a common feature of microsatellite loci, and very rare alleles are often uninformative for population‐based analyses, as their presence may be due to reoccurring mutations rather than historical association or contemporary gene flow (Hale, Burg, & Steeves, 2012). However, in this case, downstream analyses were affected by the shift in allelic variation, whereby similarities among populations were altered to become more dissimilar or more similar than observed in the “diploid” dataset. In addition, the influence of chimeras, specifically on the assessment of dispersal clusters and principal component analyses, was more pronounced for the winter samples.

Temporal sampling, while integral to ecological studies, is not always possible or is deemed too costly for genetic studies of nonindigenous species, despite multiple studies highlighting the deeper understanding of the invasion obtained from temporal sampling (Goldstien, Inglis, Schiel, & Gemmell, 2013; Pérez‐Portela et al., 2012; Pineda, Lorente, Lopez‐Legentil, Palacín, & Turon, 2016; Pineda, Turon, Pérez‐Portela, & López‐Legentil, 2016; Reem, Douek, Katzir, & Rinkevich, 2013). Pineda, Turon, et al. (2016) sampled an introduced population of the solitary ascidian *Styela plicata* before and after a summer dieback event, which had occurred for the previous 20 years since the establishment of the population. Utilizing the COI gene, they could show weak but significant differences among time periods. Specifically, the die‐off event caused an increase in genetic diversity, less evidence of inbreeding, and a net gain of microsatellite alleles. The interpretation of the full dataset suggested neighboring populations were quick to occupy the cleared space with novel alleles maintaining a viable and genetically stable population over time. In contrast to our study, Pineda, Turon, et al. (2016) consistently assigned individuals to two main genetic pools at both time periods. The assignment of individuals to populations in our study differed between sampling periods with implications to the identification of source populations.

Connectivity of populations is often inferred from genetic structure of sampled populations, and no study to our knowledge has specifically modeled and aligned dispersal clusters to genetic structure of populations within and among the clusters. Consistent with the genetic structure of sampled populations, our data were influenced by temporal sampling, whereby the winter samples better aligned to modeled clusters for an assumed 24‐hr pelagic larval duration.

This study has highlighted subtle, but important shifts in the downstream analysis of population genetic data when chimeras and seasonal dieback are considered. These shifts become more important when assessing the potential influence on management options. For instance, the assignment of source populations informs authorities of specific vector potential from an area, therefore spurious results due to sampling error may identify false introduction points and pathways of local spread, as well as inflating or understating the number of potential incursions into a newly invaded region. Similarly, the alignment of genetic structure to natural dispersal potential may divert management tools away from vector management to node clearance, yet temporal sampling indicates that this result cannot be used as conclusive if sampling is restricted to a snapshot moment.

Fletcher, Forrest, Atalah, et al. (2013) showed that *D. vexillum* reproduce for a longer period than normally considered, for 9 months of the year, but not through the winter months. They also suggest that periods of increased recruitment and growth as seen in *D. vexillum* will influence the level of connectivity and potential spread of established populations, with higher biomass leading to increased propagule pressure. Furthermore, our study suggests that the winter dieback period provides an opportunity for rare alleles to propagate through the region when growth increases again the following season, thereby suggesting that a winter removal program presents an opportunity to further reduce biomass and potentially the genetic diversity of the spreading population. While further study would be required to ensure removal does not encourage supercolonies by further reducing the genetic diversity and thereby promoting allorecognition and enhanced fusion (Smith, 2012; Smith et al., 2015), the reduction in biomass may provide enough space to boost the chances of native early colonizers.ss

Recent work by Fletcher, Forrest, and Bell (2013) indicated that natural dispersal of *D. vexillum* could exceed 1 km under high current flow regimes. Using Bayesian analyses based on these predictions and other parameters, we tested the potential for genetic connection over these distances with distinct dispersal clusters. We observed conflicting results between our summer and winter samples. The summer samples suggest genetic connectivity over 12 and 24 hr of modeled dispersal, but the winter samples were differentiated with a 24‐hr modeled period, which alone would suggest limited dispersal potential. However, in both datasets, the genetic structure was consistent with this previous study in suggesting a greater potential for long‐distance dispersal and connectivity than previously considered possible for this species (Fletcher, Forrest, & Bell, 2013).

Numerous studies have shown that in addition to natural dispersal, human activities facilitate the transport and introduction of species outside of their natural ranges (Bulleri, Balata, Bertocci, Tamburello, & Benedetti‐Cecchi, 2010; Floerl & Inglis, 2003, 2005; Floerl, Inglis, Dey, & Smith, 2009; Glasby, Connell, Holloway, & Hewitt, 2007; Johannesson & Warmoes, 1990; Lacoursière‐Roussel et al., 2012; Minchin, Floerl, Savini, & Occhipinti‐Ambrogi, 2006; Vaselli, Bulleri, & Benedetti‐Cecchi, 2008). For instance, the movements of recreational vessels have been implicated in preborder incursions by *Styela clava* in New Zealand (Goldstien et al., 2010). Acting as novel corridors or “stepping‐stones,” these structures and movements associated with human activities can generate connectivity among isolated populations, influencing genetic patterns (Airoldi et al., 2005; Fauvelot, Bertozzi, Costantini, Airoldi, & Abbiati, 2009; Fauvelot, Costantini, Virgilio, & Abbiati, 2012; Olden, LeRoy, Douglas, Douglas, & Fausch, 2004). In this study, commercial and recreational vessel movements in the Marlborough Sounds may have contributed to the observed genetic patterns and outcomes from the likelihood analyses that were unexplained by natural dispersal alone. However, as vessel movements were not explored in this paper, further investigation would be required to consider this potential dispersal impact.

Genetic data as a tool to advise management are limited by the resolution at which the organisms and their populations are sampled. Here, we have highlighted two important components of sampling and processing genetic data for colonial nonindigenous species that can alter the interpretation of genetic structure and of dispersal pathways; two primary aspects of invasion ecology that assist with the management of global and regional spread. Therefore, we would argue that seasonal sampling and the inclusion of important life history traits, such as chimerism, in genetic studies, are worth the effort for refinement of interpretations and for the management of NIS.

## CONFLICT OF INTEREST

None Declared.

## AUTHOR CONTRIBUTIONS

Ashleigh Watts conducted the work as a component of her Master's thesis under the supervision of Dr Sharyn Goldstien and Dr Grant Hopkins. All authors contributed to the design of the project. Ashleigh Watts collected and processed all genetics samples, under the guidance of Dr Sharyn Goldstien. Ashleigh Watts analyzed the data under the guidance of Dr Sharyn Goldstien and Dr Grant Hopkins. Assistance with modeling was provided by Cawthron staff.

## Data Availability

MSTAT sequences and supporting information have been submitted to Dryad.

## References

[ece34817-bib-0001] Abbott, C. L. , Ebert, D. , Tabata, A. , & Therriault, T. W. (2011). Twelve microsatellite markers in the invasive tunicate, *Didemnum vexillum*, isolated from low genome coverage 454 pyrosequencing reads. Conservation Genetics Resources, 3, 79–81. 10.1007/s12686-010-9294-2

[ece34817-bib-0002] Airoldi, L. , Abbiati, M. , Beck, M. W. , Hawkins, S. J. , Jonsson, P. R. , Martin, D. , … Åberg, P. (2005). An ecological perspective on the deployment and design of low‐crested and other hard coastal defence structures. Coastal Engineering, 52(10–11), 1073–1087. 10.1016/j.coastaleng.2005.09.007

[ece34817-bib-0003] Arif, I. A. , Khan, H. A. , Shobrak, M. , Al Homaidan, A. A. , Al Sadoon, M. , Al Farhan, A. H. , & Bahkali, A. H. (2010). Interpretation of electrophoretograms of seven microsatellite loci to determine the genetic diversity of the Arabian Oryx. Genetics and Molecular Research, 9, 259–265. 10.4238/vol9-1gmr714 20198581

[ece34817-bib-0004] Benjamini, Y. , & Yekutieli, D. (2001). The control of the false discovery rate in multiple testing under dependency. Annals of Statistics, 29, 1165–1188.

[ece34817-bib-0005] Ben‐Shlomo, R. (2017). Invasiveness, chimerism and genetic diversity. Molecular Ecology, 26, 6502–6509. 10.1111/mec.14364 28950415

[ece34817-bib-0006] Ben‐Shlomo, R. , Douek, J. , & Rinkevich, B. (2001). Heterozygote deficiency and chimerism in remote populations of a colonial ascidian from New Zealand. Marine Ecology Progress Series, 209, 109–117. 10.3354/meps209109

[ece34817-bib-0007] Bock, D. G. , Zhan, A. , Lejeusne, C. , MacIsaac, H. J. , & Cristescu, M. E. (2011). Looking at both sides of the invasion: Patterns of colonization in the violet tunicate *Botrylloides violaceus* . Molecular Ecology, 20, 503–516. 10.1111/j.1365-294X.2010.04971.x 21199029

[ece34817-bib-0008] Bullard, S. G. , Lambert, G. , Carman, M. R. , Byrnes, J. , Whitlatch, R. B. , Ruiz, G. , … Pederson, J. (2007). The colonial ascidian *Didemnum* sp. A: Current distribution, basic biology and potential threat to marine communities of the northeast and west coasts of North America. Journal of Experimental Marine Biology and Ecology, 342(1), 99–108. 10.1016/j.jembe.2006.10.020

[ece34817-bib-0009] Bulleri, F. , Balata, D. , Bertocci, I. , Tamburello, L. , & Benedetti‐Cecchi, L. (2010). The seaweed *Caulerpa racemosa* on Mediterranean rocky reefs: From passenger to driver of ecological change. Ecology, 91, 2205–2212. 10.1890/09-1857.1 20836441

[ece34817-bib-0010] Clark, L. V. , & Jasieniuk, M. (2011). polysat: An R package for polyploid microsatellite analysis. Molecular Ecology Resources, 11, 562–566. 10.1111/j.1755-0998.2011.02985.x 21481215

[ece34817-bib-0011] Coffey, B. T. (2001). Potentially invasive compound ascidian, Whangamata Harbour. Whangamata: Brian T. Coffey and Associates Limited.

[ece34817-bib-0012] Cohen, S. (1990). Outcrossing in field populations of two species of self‐fertile ascidians. Journal of Experimental Marine Biology and Ecology, 140, 147–158. 10.1016/0022-0981(90)90123-T

[ece34817-bib-0013] Eldon, B. , & Wakeley, J. (2009). Coalescence times and *F* _ST_ under a skewed offspring distribution among individuals in a population. Genetics, 181, 615–629. 10.1534/genetics.108.094342 19047415 PMC2644951

[ece34817-bib-0014] Excoffier, L. , & Lischer, H. E. (2010). Arlequin suite ver 3.5: A new series of programs to perform population genetics analyses under Linux and Windows. Molecular Ecology Resources, 10, 564–567. 10.1111/j.1755-0998.2010.02847.x 21565059

[ece34817-bib-0015] Excoffier, L. , & Slatkin, M. (1998). Incorporating genotypes of relatives into a test of linkage disequilibrium. American Journal of Human Genetics, 62, 171–180. 10.1086/301674 9443867 PMC1376801

[ece34817-bib-0016] Fauvelot, C. , Bertozzi, F. , Costantini, F. , Airoldi, L. , & Abbiati, M. (2009). Lower genetic diversity in the limpet *Patella caerulea* on urban coastal structures compared to natural rocky habitats. Marine Biology, 156, 2313–2323. 10.1007/s00227-009-1259-1

[ece34817-bib-0017] Fauvelot, C. , Costantini, F. , Virgilio, M. , & Abbiati, M. (2012). Do artificial structures alter marine invertebrate genetic makeup? Marine Biology, 159, 2797–2807. 10.1007/s00227-012-2040-4

[ece34817-bib-0018] Fletcher, L. M. , Forrest, B. M. , Atalah, J. , & Bell, J. J. (2013). Reproductive seasonality of the invasive ascidian *Didemnum vexillum* in New Zealand and implications for shellfish aquaculture. Aquaculture Environment Interactions, 3, 197–211.

[ece34817-bib-0019] Fletcher, L. M. , Forrest, B. M. , & Bell, J. J. (2013). Natural dispersal mechanisms and dispersal potential of the invasive ascidian *Didemnum vexillum* . Biological Invasions, 15, 627–643.

[ece34817-bib-0020] Floerl, O. , & Inglis, G. J. (2003). Boat harbour design can exacerbate hull fouling. Austral Ecology, 28, 116–127. 10.1046/j.1442-9993.2003.01254.x

[ece34817-bib-0021] Floerl, O. , & Inglis, G. J. (2005). Starting the invasion pathway: The interaction between source populations and human transport vectors. Biological Invasions, 7, 589–606. 10.1007/s10530-004-0952-8

[ece34817-bib-0022] Floerl, O. , Inglis, G. , Dey, K. , & Smith, A. (2009). The importance of transport hubs in stepping‐stone invasions. Journal of Applied Ecology, 46, 37–45. 10.1111/j.1365-2664.2008.01540.x

[ece34817-bib-0023] Forrest, B. M. , & Hopkins, G. A. (2013). Population control to mitigate the spread of marine pests: Insights from management of the Asian kelp *Undaria pinnatifida* and colonial ascidian *Didemnum vexillum* . Management, 4, 317–326.

[ece34817-bib-0024] García, L. V. (2004). Escaping the Bonferroni iron claw in ecological studies. Oikos, 105, 657–663. 10.1111/j.0030-1299.2004.13046.x

[ece34817-bib-0025] Gemmell, N. J. , & Akiyama, S. (1996). An efficient method for the extraction of DNA from vertebrate tissues. Trends in Genetics, 12, 338–339. 10.1016/S0168-9525(96)80005-9 8855658

[ece34817-bib-0026] Gerlach, G. , Jueterbock, A. , Kraemer, P. , Deppermann, J. , & Harmand, P. (2010). Calculations of population differentiation based on GST and D: Forget GST but not all of statistics!. Molecular Ecology, 19, 3845–3852. 10.1111/j.1365-294X.2010.04784.x 20735737

[ece34817-bib-0027] Glasby, T. , Connell, S. , Holloway, M. , & Hewitt, C. (2007). Nonindigenous biota on artificial structures: Could habitat creation facilitate biological invasions? Marine Biology, 151, 887–895. 10.1007/s00227-006-0552-5

[ece34817-bib-0028] Goldstien, S. J. , Inglis, G. J. , Schiel, D. R. , & Gemmell, N. J. (2013). Using temporal sampling to improve attribution of source populations for invasive species. PLoS One, 8, e65656 10.1371/journal.pone.0065656 23755264 PMC3670837

[ece34817-bib-0029] Goldstien, S. , Schiel, D. , & Gemmell, N. (2010). Regional connectivity and coastal expansion: Differentiating pre‐border and post‐border vectors for the invasive tunicate *Styela clava* . Molecular Ecology, 19, 874–885. 20149095 10.1111/j.1365-294X.2010.04527.x

[ece34817-bib-0030] Hale, M. L. , Burg, T. M. , & Steeves, T. E. (2012). Sampling for microsatellite‐based population genetic studies: 25 to 30 individuals per population is enough to accurately estimate allele frequencies. PLoS ONE, 7, e45170 10.1371/journal.pone.0045170 22984627 PMC3440332

[ece34817-bib-0031] Herborg, L.‐M. , O'Hara, P. , & Therriault, T. W. (2009). Forecasting the potential distribution of the invasive tunicate *Didemnum vexillum* . Journal of Applied Ecology, 46, 64–72.

[ece34817-bib-0032] Hess, J. E. , Swalla, B. J. , & Moran, P. (2009). New molecular markers to genetically differentiate populations of *Didemnum vexillum* (Kott, 2002)–an invasive ascidian species. Aquatic Invasions, 4, 299–310. 10.3391/ai.2009.4.2.1

[ece34817-bib-0033] Holleley, C. E. , & Geerts, P. G. (2009). Multiplex Manager 1.0: A cross‐platform computer program that plans and optimizes multiplex PCR. BioTechniques, 46, 511–517. 10.2144/000113156 19594450

[ece34817-bib-0034] Johannesson, K. , & Warmoes, T. (1990). Rapid colonization of Belgian breakwaters by the direct developer, *Littorina saxatilis* (Olivi) (Prosobranchia, Mollusca) In Johannesson K., Raffaelli D. G., & Hannaford Ellis C. J. (Eds.), Progress in Littorinid and Muricid Biology (pp. 99–108). Dordrecht: Springer.

[ece34817-bib-0035] Jost, L. (2008). GST and its relatives do not measure differentiation. Molecular Ecology, 17, 4015–4026. 19238703 10.1111/j.1365-294x.2008.03887.x

[ece34817-bib-0036] Jueterbock, A. , Kraemer, P. , Gerlach, G. , & Deppermann, J. (2011). DEMEtics: Evaluating the genetic differentiation between populations based on GST and D values. R package, version 0.8‐2.8.

[ece34817-bib-0037] Jueterbock, A. , Kraemer, P. , Gerlach, G. , Deppermann, J. , & Jueterbock, M. A. (2013). Package ‘DEMEtics’. Molecular Ecology, 19, 3845–3852. 10.1111/j.1365-294X.2010.04784.x 20735737

[ece34817-bib-0038] Knight, B. , & Beamsley, B. (2012) Calibration and methodology report for hydrodynamic models of the Marlborough Sounds. Cawthron Report 2028. Prepared for New Zealand King Salmon Company Limited.

[ece34817-bib-0039] Lacoursière‐roussel, A. , Bock, D. G. , Cristescu, M. E. , Guichard, F. , Girard, P. , Legendre, P. , & McKindsey, C. W. (2012). Disentangling invasion processes in a dynamic shipping–boating network. Molecular Ecology, 21, 4227–4241. 10.1111/j.1365-294X.2012.05702.x 22804778

[ece34817-bib-0040] McDonald, S. , & Acosta, H. (2012). New marine biosecurity information system goes online. Ministry for Primary Industries. http://www.marinebiosecurity.org.nz/project-map-all-data/?species=Didemnum%20vexillum&bbox=164.323125,-47.976015625,185.416875,-35.143984375.

[ece34817-bib-0041] Minchin, D. , Floerl, O. , Savini, D. , & Occhipinti‐Ambrogi, A. (2006). Small craft and the spread of exotic species In: Davenport J. & Davenport J. D. (Eds.), The ecology of transportation: Managing mobility for the environment (pp. 99–118). Dordrecht, The Netherlands: Springer.

[ece34817-bib-0042] Morris, J. A. , & Carman, M. R. (2012). Fragment reattachment, reproductive status, and health indicators of the invasive colonial tunicate *Didemnum vexillum* with implications for dispersal. Biological Invasions, 14, 2133–2140. 10.1007/s10530-012-0219-8

[ece34817-bib-0043] Narum, S. R. (2006). Beyond Bonferroni: Less conservative analyses for conservation genetics. Conservation Genetics, 7, 783–787. 10.1007/s10592-005-9056-y

[ece34817-bib-0044] Olden, J. D. , LeRoy, P. N. , Douglas, M. R. , Douglas, M. E. , & Fausch, K. D. (2004). Ecological and evolutionary consequences of biotic homogenization. Trends in Ecology & Evolution, 19, 18–24. 10.1016/j.tree.2003.09.010 16701221

[ece34817-bib-0045] Osman, R. W. , & Whitlatch, R. B. (2007). Variation in the ability of *Didemnum* sp. to invade established communities. Journal of Experimental Marine Biology and Ecology, 342, 40–53. 10.1016/j.jembe.2006.10.013

[ece34817-bib-0046] Papacostas, K. J. , Rielly‐Carroll, E. W. , Georgian, S. E. , Long, D. J. , Princiotta, S. D. , Quattrini, A. M. , … Freestone, A. L. (2017). Biological mechanisms of marine invasions. Marine Ecology Progress Series, 565, 251–268. 10.3354/meps12001

[ece34817-bib-0047] Pérez‐Portela, R. , Turon, X. , & Bishop, J. D. D. (2012). Bottlenecks and loss of genetic diversity: Spatio‐temporal patterns of genetic structure in an ascidian recently introduced in Europe. Marine Ecology Progress Series, 451, 93–105. 10.3354/meps09560

[ece34817-bib-0048] Pineda, M.‐C. , Lorente, B. , Lopez‐Legentil, S. , Palacín, C. , & Turon, X. (2016). Stochasticity in space, persistence in time: Genetic heterogeneity in harbour populations of the introduced ascidian *Styela plicata* . PeerJ, 4, e2158. 27366653 10.7717/peerj.2158 PMC4924124

[ece34817-bib-0049] Pineda, M. C. , Turon, X. , Pérez‐Portela, R. , & López‐Legentil, S. (2016). Stable populations in unstable habitats: Temporal genetic structure of the introduced ascidian *Styela plicata* in North Carolina. Marine Biology, 163, 59.

[ece34817-bib-0050] Pritchard, J. K. , Stephens, M. , & Donnelly, P. (2000). Inference of population structure using multilocus genotype data. Genetics, 155, 945–959. 10835412 10.1093/genetics/155.2.945 PMC1461096

[ece34817-bib-0051] R Core Team (2013). R: A language and environment for statistical computing. Vienna, Austria: R Foundation for Statistical Computing.

[ece34817-bib-0052] Reem, E. , Douek, J. , Katzir, G. , & Rinkevich, B. (2013). Long‐term population genetic structure of an invasive urochordate: The ascidian *Botryllus schlosseri* . Biological Invasions, 15, 225–241. 10.1007/s10530-012-0281-2

[ece34817-bib-0053] Sakai, A. K. , Allendorf, F. W. , Holt, J. S. , Lodge, D. M. , Molofsky, J. , With, K. A. , … McCauley, D. E. (2001). The population biology of invasive species. Annual Review of Ecology and Systematics, 32(1), 305–332. 10.1146/annurev.ecolsys.32.081501.114037

[ece34817-bib-0054] Schuelke, M. (2000). An economic method for the fluorescent labeling of PCR fragments. Nature Biotechnology, 18, 233–234. 10.1038/72708 10657137

[ece34817-bib-0055] Sheets, E. A. , Cohen, C. S. , Ruiz, G. M. , & Rocha, R. M. (2016). Investigating the widespread introduction of a tropical marine fouling species. Ecology and Evolution, 6, 2453–2471. 10.1002/ece3.2065 27066231 PMC4788974

[ece34817-bib-0056] Smith, K. F. (2012). Use of genetic methods for determining patterns and processes during marine biological invasions. PhD Thesis, University of Waikato.

[ece34817-bib-0057] Smith, K. F. , Abbott, C. L. , Saito, Y. , & Fidler, A. E. (2015). Comparison of whole mitochondrial genome sequences from two clades of the invasive ascidian, *Didemnum vexillum* . Marine Genomics, 19, 75–83. 10.1016/j.margen.2014.11.007 25482898

[ece34817-bib-0058] Smith, K. F. , Stefaniak, L. , Saito, Y. , Gemmill, C. E. C. , Cary, S. C. , & Fidler, A. E. (2012). Increased inter‐colony fusion rates are associated with reduced COI haplotype diversity in an invasive colonial ascidian *Didemnum vexillum* . PLoS One, 7, e30473 10.1371/journal.pone.0030473 22303442 PMC3269411

[ece34817-bib-0059] Valentine, P. C. , Carman, M. R. , Blackwood, D. S. , & Heffron, E. J. (2007). Ecological observations on the colonial ascidian *Didemnum* sp. in a New England tide pool habitat. Journal of Experimental Marine Biology and Ecology, 342, 109–121. 10.1016/j.jembe.2006.10.021

[ece34817-bib-0060] Van Oosterhout, C. , Hutchinson, W. F. , Wills, D. P. , & Shipley, P. (2004). MICRO‐CHECKER: Software for identifying and correcting genotyping errors in microsatellite data. Molecular Ecology Notes, 4, 535–538. 10.1111/j.1471-8286.2004.00684.x

[ece34817-bib-0061] Vaselli, S. , Bulleri, F. , & Benedetti‐Cecchi, L. (2008). Hard coastal‐defence structures as habitats for native and exotic rocky‐bottom species. Marine Environmental Research, 66, 395–403. 10.1016/j.marenvres.2008.06.002 18656256

[ece34817-bib-0062] Watts, A. M. (2014). Biofouling patterns and local dispersal in an aquaculture system in the Marlborough Sounds, New Zealand. MSc Thesis, University of Canterbury.

[ece34817-bib-0063] Wright, S. (1965). The interpretation of population structure by F‐statistics with special regard to systems of mating. Evolution, 19, 395–420. 10.1111/j.1558-5646.1965.tb01731.x

[ece34817-bib-0064] Wright, S. (1978) Evolution and the genetics of populations. Vol. 4: Variability within and among natural populations. Chicago, IL: University of Chicago Press.

[ece34817-bib-0065] Zhan, A. , Darling, J. A. , Bock, D. G. , Lacoursière‐Roussel, A. , MacIsaac, H. J. , & Cristescu, M. E. (2012). Complex genetic patterns in closely related colonising invasive species. Ecology and Evolution, 2, 1331–1346. 22957143 10.1002/ece3.258 PMC3434944

[ece34817-bib-0066] Zhu, S. , Degnan, J. H. , Goldstien, S. J. , & Eldon, B. (2015). Hybrid‐Lambda: Simulation of multiple merger and Kingman gene genealogies in species networks and species trees. BMC Bioinformatics, 16, 292 10.1186/s12859-015-0721-y 26373308 PMC4571064

