# Supplementary material for: Chimerism and population dieback alter genetic inference related to invasion pathways and connectivity of biofouling populations on artificial substrata
Source: Ecol Evol. 2019 Feb 21;9(6):3089–104. doi: 10.1002/ece3.4817 (PMC6434572; doi:10.1002/ece3.4817)
Supplement: Supplementary file 1 [file ECE3-9-3089-s001.docx]

**Supporting Information for Ecology and Evolution publication by Watts *et al*., 2018.**

Supplementary Tables

Table S1. Characterisation of eight primer pairs amplifying DNA microsatellites in *Didemnum vexillum*. Each locus name, the sequences for the locus-specific M13-labelled forward and reverse primers, repeat motifs, locus-specific allele sizes, allele ranges, M13 label for multiplexing, multiplex groups and paired loci (note that only details for the paired loci that consistently worked and are included in the final analysis are given in detail, i.e., the eight primers, those highlighted in bold did not work consistently across all populations and are not included in the final analyses), the number of alleles for each locus in winter and summer (AN) and the expected heterozygosity (HE) under Hardy-Weinberg for winter and summer are all displayed. All means ± SE.

| **Locus** | **Primer sequence (5’-3’)** | **Repeat Motif** | **Allele sizes (bp)** | **Allele Range (bp)** | **M13 label** | **Multiplex group and loci pair** | **AN**  **(summer)** | **AN (winter)** | **HE (±S.E)**  **(summer)** | **HE (±S.E)**  **(winter)** |
| --- | --- | --- | --- | --- | --- | --- | --- | --- | --- | --- |
| DVEX10 | F: M13-TTGGAAGTGCTTTGGTAGCC | (ACA)7 | 159-201 | 159-201 | FAM | Group1, multiplexed with **DVEX26** | 8 | 6 | 0.70 (± 0.01) | 0.67 (±0.02) |
|  | R: TGCCAATAGTCAGGTTTGTCG |  |  |  |  |  |  |  |  |  |
| DVEX01 | F: M13-CCAACCATGAGTGTGAAGCG | (AGT)7 | 74-116 | 74-119 | PET | Group1, multiplexed with  **DVEX14** | 4 | 3 | 0.47 (± 0.01) | 0.43 (±0.05) |
|  | R: GCGTTACTGTTGATTGAAGCC |  |  |  |  |  |  |  |  |  |
| DVEX19 | F: M13-CTGGTCCAAATAACGAACGATTG | (CACG)7 | 211-267 | 211-267 | FAM | Group2, multiplexed with  **DVEX37** | 9 | 8 | 0.70 (± 0.02) | 0.64 (±0.03) |
|  | R: TTTACGAGCAGCCAACGAAC |  |  |  |  |  |  |  |  |  |
| DVEX11 | F: M13-TCAGGGCCCCAAATACCAAG | (AC)12 | 167-215 | 167-215 | PET | Group2, not multiplexed | 9 | 6 | 0.63 (± 0.02) | 0.52 (±0.06) |
|  | R: ACCATAACCCTAGAACATACCC |  |  |  |  |  |  |  |  |  |
| DVEX36 | F: M13-TGTTACTCATGCACTTGCGG | (CAA)7 | 145-187 | 145-187 | VIC | Group2, multiplexed with  DVEX30 | 9 | 7 | 0.52 (± 0.01) | 0.60 (±0.04) |
|  | R: TGCATTGGTTCGACCTGTTG |  |  |  |  |  |  |  |  |  |
| DVEX30 | F: M13-TTCATCCGGGTACTCGACAG | (AGA)7 | 211-253 | 211-253 | VIC | Group2, multiplexed with  DVEX36 | 13 | 8 | 0.56 (± 0.03) | 0.48 (±0.07) |
|  | R: GGGTCTTGGGCGTGTTTATG |  |  |  |  |  |  |  |  |  |
| DVEX33 | F: M13-GGAACGGATGATGATGGCTG | (GTT)8 | 105-153 | 105-153 | NED | Group2, multiplexed with  DVEX03 | 13 | 8 | 0.76 (± 0.01) | 0.72 (±0.03) |
|  | R: GCAGTCTTGTCAGGGAGGAG |  |  |  |  |  |  |  |  |  |
| DVEX03 | F: M13-TGTTTCAGGCGAGTTCATCG | (TGA)7 | 196-238 | 196-238 | NED | Group2, multiplexed with  DVEX33 | 10 | 9 | 0.47 (± 0.03) | 0.45 (±0.08) |
|  | R: CAATGAACAAACGCAACCGC |  |  |  |  |  |  |  |  |  |
| M13 | GTAAAACGACGGCCAGT |  |  |  |  |  |  |  |  |  |
| **Mean** |  |  |  |  |  |  | 9.38 | 6.88 | 0.60 (± 0.04) | 0.56 (±0.01) |

Table S2. Pairwise FST (below diagonal) and Jost’s D (above diagonal) matrix (including all loci) for each cluster grouping for summer populations in Pelorus Sound, from 24-hour (a), to 12-hour (b) and 2-hour clusters (c). Significant pairwise combinations after False Discovery Rate corrections for multiple tests (N=36) are indicated in bold, P ≤ 0.001. Pelorus Sound populations include: GOUL=Goulter Bay, SCHN=Schnapper Point, HIKA=Hikapu Reach, NYDA=Nydia Bay, YNCY=Yncyca Bay, TAWO=Tawero Point, HALM= Hallam Cove, FORS=Forsyth Bay and MELV=Melville Cove.

| 1. **24-hour PLD** | |  |  |  |  |  |  |  |  |
| --- | --- | --- | --- | --- | --- | --- | --- | --- | --- |
| *Clust 1* | | *Clust 2* | | | | | | | *Clust 3* |
|  | GOUL | SCHN | HIKA | NYDA | YNCY | TAWO | HALM | FORS | MELV |
| GOUL | - | 0.05 | **0.09** | **0.11** | **0.10** | 0.03 | 0.05 | 0.05 | 0.03 |
| SCHN | 0.01 | - | **0.06** | 0.03 | 0.00 | **0.07** | **0.08** | **0.14** | 0.05 |
| HIKA | **0.05** | 0.02 | - | 0.00 | **0.08** | **0.09** | **0.06** | **0.15** | **0.08** |
| NYDA | **0.05** | **0.01** | 0.00 | - | 0.04 | **0.14** | **0.11** | **0.20** | **0.11** |
| YNCY | **0.04** | 0.00 | **0.04** | 0.02 | - | **0.15** | **0.11** | **0.18** | **0.12** |
| TAWO | 0.01 | **0.03** | **0.04** | **0.06** | **0.06** | - | 0.06 | **0.08** | 0.04 |
| HALM | 0.01 | 0.02 | 0.02 | **0.04** | **0.04** | **0.02** | - | 0.01 | 0.05 |
| FORS | **0.03** | **0.07** | **0.09** | **0.10** | **0.10** | **0.05** | 0.00 | - | **0.06** |
| MELV | 0.02 | 0.02 | **0.04** | **0.05** | **0.06** | 0.02 | 0.01 | **0.03** | - |
|  |  |  |  |  |  |  | - |  |  |
|  |  |  |  |  |  |  |  |  |  |
| 1. **12-hour PLD** | |  |  |  |  |  |  |  |  |
| *Clust 1* | | *Clust 2* | | | | | | *Clust 3* | *Clust 4* |
|  | GOUL | SCHN | HIKA | NYDA | YNCY | TAWO | FORS | HALM | MELV |
| GOUL | - | 0.05 | **0.09** | **0.11** | **0.10** | 0.03 | 0.05 | 0.05 | 0.03 |
| SCHN | 0.01 | - | **0.06** | 0.03 | 0.00 | **0.07** | **0.14** | **0.08** | 0.05 |
| HIKA | **0.05** | 0.02 | - | 0.00 | **0.08** | **0.09** | **0.15** | **0.06** | **0.08** |
| NYDA | **0.05** | **0.01** | 0.00 | - | 0.04 | **0.14** | **0.20** | **0.11** | **0.11** |
| YNCY | **0.04** | 0.00 | **0.04** | 0.02 | - | **0.15** | **0.18** | **0.11** | **0.12** |
| TAWO | 0.01 | **0.03** | **0.04** | **0.06** | **0.06** | - | **0.08** | 0.06 | 0.04 |
| FORS | 0.03 | **0.07** | **0.09** | **0.10** | **0.10** | **0.05** | - | 0.01 | 0.05 |
| HALM | **0.01** | 0.02 | **0.02** | **0.04** | **0.04** | **0.02** | 0.01 | - | **0.06** |
| MELV | 0.02 | 0.02 | **0.04** | **0.05** | **0.06** | 0.02 | 0.03 | 0.01 | - |
|  |  |  |  |  |  |  |  |  |  |
|  |  |  |  |  |  |  |  |  |  |
| 1. **2-hour PLD** | |  |  |  |  |  |  |  |  |
| *Clust 1* | | *Clust 2* | *Clust 3* | *Clust 4* | *Cluster 5* | *Clust 6* | *Clust 7* | *Clust 8* | *Clust 9* |
|  | GOUL | SCHN | HIKA | NYDA | YNCY | TAWO | FORS | HALM | MELV |
| GOUL | - | 0.05 | **0.09** | **0.11** | **0.10** | 0.03 | **0.05** | 0.05 | 0.03 |
| SCHN | 0.01 | - | **0.06** | 0.03 | 0.00 | **0.07** | **0.14** | **0.08** | 0.05 |
| HIKA | **0.05** | 0.02 | - | 0.00 | **0.08** | **0.09** | **0.15** | **0.06** | **0.08** |
| NYDA | **0.05** | **0.01** | 0.00 | - | 0.04 | **0.14** | **0.20** | **0.11** | **0.11** |
| YNCY | **0.04** | 0.00 | **0.04** | 0.02 | - | **0.15** | **0.18** | **0.11** | **0.12** |
| TAWO | 0.01 | **0.03** | **0.04** | **0.06** | **0.06** | - | **0.08** | 0.06 | 0.04 |
| FORS | **0.03** | **0.07** | **0.09** | **0.10** | **0.10** | **0.05** | - | 0.01 | 0.05 |
| HALM | 0.01 | 0.02 | 0.02 | **0.04** | **0.04** | **0.02** | 0.01 | - | **0.06** |
| MELV | 0.02 | 0.02 | **0.04** | **0.05** | **0.06** | 0.02 | **0.03** | 0.01 | - |
|  |  |  |  |  |  |  |  |  |  |
|  |  |  |  |  |  |  |  |  |  |

Table S3. Allele frequencies for each loci within each population for the diploid summer dataset. Bold values denote alleles with different frequencies to those attained using the polyploid dataset (polyploid dataset found in Table S4).

|  | | |  |  |  |  |  |  |  |  |  |  |  |  |  |  |  |
| --- | --- | --- | --- | --- | --- | --- | --- | --- | --- | --- | --- | --- | --- | --- | --- | --- | --- |
| **DIPLOID_Summer** | | |  |  |  |  |  |  |  |  |  |  |  |  |  |  |  |
| **Locus** | **Allele** | **Goulter** | **Schnapper** | **Hikapu** | **Nydia** | **Yncyca** | **Tawero** | **Hallam** | **Forsyth** | **Melville** | **Picton** | **Shakespeare** | **Onahau** | **Ruakaka** | **Te Aroha** | **Port Nelson** | ***Average*** |
| DVEX10 | 174 | 0.00 | 0.00 | 0.00 | 0.00 | 0.00 | 0.00 | 0.00 | 0.02 | 0.00 | 0.00 | 0.00 | 0.00 | 0.00 | 0.00 | 0.00 | 0.00 (± 0.00) |
|  | **177** | **0.02** | **0.13** | **0.12** | **0.13** | **0.13** | **0.17** | **0.02** | **0.00** | **0.11** | **0.09** | **0.02** | **0.03** | **0.09** | **0.08** | **0.02** | **0.08 (± 0.01)** |
|  | 180 | 0.10 | 0.15 | 0.10 | 0.13 | 0.04 | 0.11 | 0.14 | 0.07 | 0.13 | 0.07 | 0.08 | 0.13 | 0.11 | 0.02 | 0.00 | 0.09 (± 0.01) |
|  | 183 | 0.33 | 0.21 | 0.26 | 0.22 | 0.29 | 0.22 | 0.31 | 0.41 | 0.20 | 0.20 | 0.26 | 0.32 | 0.29 | 0.25 | 0.43 | 0.28 (± 0.02) |
|  | 186 | 0.06 | 0.15 | 0.10 | 0.12 | 0.15 | 0.13 | 0.10 | 0.04 | 0.11 | 0.11 | 0.08 | 0.03 | 0.05 | 0.10 | 0.07 | 0.09 (± 0.01) |
|  | 189 | 0.00 | 0.00 | 0.00 | 0.02 | 0.00 | 0.00 | 0.00 | 0.00 | 0.00 | 0.00 | 0.00 | 0.00 | 0.00 | 0.00 | 0.02 | 0.00 (± 0.00) |
|  | **192** | **0.38** | **0.35** | **0.40** | **0.38** | **0.37** | **0.31** | **0.43** | **0.46** | **0.44** | **0.52** | **0.54** | **0.50** | **0.45** | **0.50** | **0.43** | **0.43 (± 0.02)** |
|  | **195** | **0.10** | **0.02** | **0.02** | **0.00** | **0.02** | **0.06** | **0.00** | **0.00** | **0.00** | **0.02** | **0.02** | **0.00** | **0.02** | **0.04** | **0.02** | **0.02 (± 0.01)** |
|  |  |  |  |  |  |  |  |  |  |  |  |  |  |  |  |  |  |
| DVEX01 | 106 | 0.32 | 0.41 | 0.23 | 0.27 | 0.38 | 0.35 | 0.50 | 0.54 | 0.33 | 0.44 | 0.48 | 0.47 | 0.50 | 0.40 | 0.43 | 0.40 (± 0.02) |
|  | 112 | 0.68 | 0.59 | 0.77 | 0.71 | 0.62 | 0.65 | 0.50 | 0.42 | 0.66 | 0.56 | 0.52 | 0.53 | 0.50 | 0.60 | 0.58 | 0.59 (± 0.02) |
|  | 115 | 0.00 | 0.00 | 0.00 | 0.00 | 0.00 | 0.00 | 0.00 | 0.04 | 0.00 | 0.00 | 0.00 | 0.00 | 0.00 | 0.00 | 0.00 | 0.00 (± 0.00) |
|  | 118 | 0.00 | 0.00 | 0.00 | 0.02 | 0.00 | 0.00 | 0.00 | 0.00 | 0.02 | 0.00 | 0.00 | 0.00 | 0.00 | 0.00 | 0.00 | 0.00 (± 0.00) |
|  |  |  |  |  |  |  |  |  |  |  |  |  |  |  |  |  |  |
| DVEX19 | 235 | 0.00 | 0.00 | 0.00 | 0.00 | 0.00 | 0.00 | 0.00 | 0.04 | 0.00 | 0.00 | 0.00 | 0.00 | 0.00 | 0.00 | 0.00 | 0.00 (± 0.00) |
|  | 239 | 0.10 | 0.02 | 0.00 | 0.00 | 0.04 | 0.11 | 0.03 | 0.06 | 0.09 | 0.03 | 0.21 | 0.28 | 0.07 | 0.09 | 0.12 | 0.08 (± 0.02) |
|  | 243 | 0.54 | 0.40 | 0.24 | 0.21 | 0.35 | 0.35 | 0.42 | 0.52 | 0.33 | 0.31 | 0.44 | 0.06 | 0.50 | 0.48 | 0.35 | 0.37 (± 0.03) |
|  | 247 | 0.10 | 0.21 | 0.56 | 0.57 | 0.29 | 0.04 | 0.34 | 0.08 | 0.17 | 0.09 | 0.08 | 0.06 | 0.00 | 0.04 | 0.03 | 0.18 (± 0.05) |
|  | 251 | 0.08 | 0.08 | 0.15 | 0.19 | 0.04 | 0.13 | 0.13 | 0.19 | 0.17 | 0.13 | 0.19 | 0.50 | 0.27 | 0.22 | 0.29 | 0.18 (± 0.03) |
|  | 255 | 0.18 | 0.21 | 0.04 | 0.03 | 0.12 | 0.28 | 0.03 | 0.10 | 0.24 | 0.16 | 0.06 | 0.00 | 0.13 | 0.11 | 0.00 | 0.11 (± 0.02) |
|  | 259 | 0.00 | 0.00 | 0.00 | 0.00 | 0.04 | 0.04 | 0.00 | 0.00 | 0.00 | 0.00 | 0.00 | 0.00 | 0.00 | 0.00 | 0.00 | 0.01 (± 0.00) |
|  | 263 | 0.00 | 0.04 | 0.02 | 0.00 | 0.13 | 0.04 | 0.05 | 0.02 | 0.00 | 0.28 | 0.02 | 0.11 | 0.04 | 0.02 | 0.21 | 0.07 (± 0.02) |
|  | 267 | 0.00 | 0.04 | 0.00 | 0.00 | 0.00 | 0.02 | 0.00 | 0.00 | 0.00 | 0.00 | 0.00 | 0.00 | 0.00 | 0.04 | 0.00 | 0.01 (± 0.00) |
|  |  |  |  |  |  |  |  |  |  |  |  |  |  |  |  |  |  |
| DVEX11 | 179 | 0.00 | 0.00 | 0.00 | 0.00 | 0.00 | 0.02 | 0.00 | 0.00 | 0.00 | 0.00 | 0.00 | 0.00 | 0.00 | 0.00 | 0.00 | 0.00 (± 0.00) |
|  | 195 | 0.06 | 0.08 | 0.07 | 0.15 | 0.02 | 0.13 | 0.05 | 0.04 | 0.02 | 0.02 | 0.06 | 0.08 | 0.02 | 0.06 | 0.07 | 0.06 (± 0.01) |
|  | **197** | **0.12** | **0.13** | **0.07** | **0.07** | **0.14** | **0.02** | **0.07** | **0.06** | **0.07** | **0.09** | **0.10** | **0.06** | **0.02** | **0.04** | **0.31** | **0.09 (± 0.02)** |
|  | 201 | 0.00 | 0.00 | 0.00 | 0.00 | 0.00 | 0.00 | 0.00 | 0.00 | 0.00 | 0.02 | 0.00 | 0.00 | 0.00 | 0.00 | 0.00 | 0.00 (± 0.00) |
|  | 205 | 0.08 | 0.03 | 0.04 | 0.02 | 0.06 | 0.02 | 0.02 | 0.08 | 0.06 | 0.04 | 0.06 | 0.00 | 0.20 | 0.08 | 0.00 | 0.05 (± 0.01) |
|  | **207** | **0.31** | **0.43** | **0.64** | **0.52** | **0.44** | **0.57** | **0.52** | **0.38** | **0.44** | **0.50** | **0.29** | **0.72** | **0.28** | **0.27** | **0.55** | **0.46 (± 0.04)** |
|  | 213 | 0.00 | 0.00 | 0.00 | 0.00 | 0.02 | 0.00 | 0.00 | 0.00 | 0.00 | 0.00 | 0.00 | 0.00 | 0.00 | 0.00 | 0.00 | 0.00 (± 0.00) |
|  | 215 | 0.04 | 0.00 | 0.00 | 0.00 | 0.00 | 0.00 | 0.00 | 0.08 | 0.00 | 0.00 | 0.02 | 0.00 | 0.00 | 0.00 | 0.05 | 0.01 (± 0.01) |
|  | **217** | **0.40** | **0.35** | **0.18** | **0.24** | **0.32** | **0.24** | **0.33** | **0.37** | **0.41** | **0.33** | **0.48** | **0.14** | **0.48** | **0.54** | **0.02** | **0.32 (± 0.04)** |
|  |  |  |  |  |  |  |  |  |  |  |  |  |  |  |  |  |  |
| DVEX36 | 142 | 0.00 | 0.00 | 0.00 | 0.00 | 0.00 | 0.00 | 0.00 | 0.02 | 0.00 | 0.00 | 0.00 | 0.08 | 0.00 | 0.00 | 0.00 | 0.01 (± 0.01) |
|  | 151 | 0.00 | 0.00 | 0.00 | 0.02 | 0.00 | 0.00 | 0.00 | 0.00 | 0.00 | 0.00 | 0.00 | 0.00 | 0.02 | 0.00 | 0.00 | 0.00 (± 0.00) |
|  | 154 | 0.00 | 0.00 | 0.00 | 0.00 | 0.00 | 0.00 | 0.00 | 0.02 | 0.05 | 0.02 | 0.00 | 0.00 | 0.00 | 0.00 | 0.00 | 0.01 (± 0.00) |
|  | 163 | 0.00 | 0.00 | 0.00 | 0.00 | 0.00 | 0.00 | 0.00 | 0.00 | 0.02 | 0.00 | 0.00 | 0.00 | 0.00 | 0.00 | 0.00 | 0.00 (± 0.00) |
|  | 166 | 0.25 | 0.31 | 0.34 | 0.31 | 0.19 | 0.37 | 0.21 | 0.20 | 0.24 | 0.21 | 0.29 | 0.18 | 0.13 | 0.26 | 0.23 | 0.25 (± 0.02) |
|  | 169 | 0.02 | 0.08 | 0.13 | 0.10 | 0.10 | 0.06 | 0.12 | 0.07 | 0.02 | 0.02 | 0.08 | 0.08 | 0.07 | 0.13 | 0.10 | 0.08 (± 0.01) |
|  | 172 | 0.00 | 0.00 | 0.00 | 0.00 | 0.00 | 0.00 | 0.00 | 0.00 | 0.00 | 0.00 | 0.02 | 0.00 | 0.02 | 0.00 | 0.00 | 0.00 (± 0.00) |
|  | 175 | 0.62 | 0.58 | 0.54 | 0.57 | 0.71 | 0.56 | 0.67 | 0.70 | 0.67 | 0.69 | 0.62 | 0.66 | 0.73 | 0.61 | 0.60 | 0.63 (± 0.02) |
|  | 178 | 0.12 | 0.02 | 0.00 | 0.00 | 0.00 | 0.02 | 0.00 | 0.00 | 0.00 | 0.06 | 0.00 | 0.00 | 0.04 | 0.00 | 0.06 | 0.02 (± 0.01) |
|  |  |  |  |  |  |  |  |  |  |  |  |  |  |  |  |  |  |
| DVEX30 | 211 | 0.00 | 0.00 | 0.00 | 0.00 | 0.00 | 0.00 | 0.00 | 0.00 | 0.04 | 0.00 | 0.00 | 0.00 | 0.00 | 0.00 | 0.00 | 0.00 (± 0.00) |
|  | 214 | 0.00 | 0.05 | 0.00 | 0.08 | 0.00 | 0.00 | 0.06 | 0.00 | 0.02 | 0.00 | 0.06 | 0.00 | 0.00 | 0.00 | 0.00 | 0.02 (± 0.01) |
|  | 229 | 0.00 | 0.00 | 0.00 | 0.00 | 0.00 | 0.00 | 0.00 | 0.00 | 0.00 | 0.00 | 0.00 | 0.00 | 0.00 | 0.00 | 0.02 | 0.00 (± 0.00) |
|  | 232 | 0.02 | 0.00 | 0.00 | 0.00 | 0.00 | 0.00 | 0.00 | 0.00 | 0.00 | 0.00 | 0.02 | 0.00 | 0.00 | 0.00 | 0.00 | 0.00 (± 0.00) |
|  | 235 | 0.02 | 0.03 | 0.02 | 0.06 | 0.00 | 0.00 | 0.00 | 0.00 | 0.05 | 0.00 | 0.00 | 0.00 | 0.04 | 0.02 | 0.00 | 0.02 (± 0.01) |
|  | 238 | 0.13 | 0.29 | 0.30 | 0.23 | 0.13 | 0.30 | 0.09 | 0.16 | 0.14 | 0.00 | 0.10 | 0.06 | 0.13 | 0.12 | 0.02 | 0.15 (± 0.02) |
|  | 241 | 0.21 | 0.03 | 0.02 | 0.00 | 0.02 | 0.14 | 0.03 | 0.02 | 0.00 | 0.40 | 0.18 | 0.22 | 0.05 | 0.08 | 0.54 | 0.13 (± 0.04) |
|  | 244 | 0.00 | 0.00 | 0.04 | 0.00 | 0.00 | 0.00 | 0.00 | 0.02 | 0.04 | 0.00 | 0.00 | 0.06 | 0.00 | 0.00 | 0.00 | 0.01 (± 0.00) |
|  | 247 | 0.04 | 0.08 | 0.00 | 0.00 | 0.00 | 0.00 | 0.00 | 0.02 | 0.11 | 0.02 | 0.02 | 0.00 | 0.00 | 0.00 | 0.02 | 0.02 (± 0.01) |
|  | 250 | 0.54 | 0.42 | 0.52 | 0.54 | 0.56 | 0.54 | 0.78 | 0.78 | 0.39 | 0.58 | 0.54 | 0.67 | 0.77 | 0.75 | 0.40 | 0.58 (± 0.03) |
|  | 253 | 0.04 | 0.00 | 0.00 | 0.00 | 0.04 | 0.02 | 0.00 | 0.00 | 0.05 | 0.00 | 0.08 | 0.00 | 0.00 | 0.04 | 0.00 | 0.02 (± 0.01) |
|  | 256 | 0.00 | 0.00 | 0.00 | 0.00 | 0.00 | 0.00 | 0.00 | 0.00 | 0.02 | 0.00 | 0.00 | 0.00 | 0.00 | 0.00 | 0.00 | 0.00 (± 0.00) |
|  | 259 | 0.00 | 0.11 | 0.11 | 0.10 | 0.25 | 0.00 | 0.03 | 0.00 | 0.14 | 0.00 | 0.00 | 0.00 | 0.02 | 0.00 | 0.00 | 0.05 (± 0.02) |
|  |  |  |  |  |  |  |  |  |  |  |  |  |  |  |  |  |  |
| DVEX33 | 105 | 0.00 | 0.00 | 0.00 | 0.02 | 0.00 | 0.00 | 0.00 | 0.00 | 0.00 | 0.00 | 0.00 | 0.00 | 0.00 | 0.00 | 0.00 | 0.00 (± 0.00) |
|  | 111 | 0.00 | 0.00 | 0.00 | 0.00 | 0.00 | 0.00 | 0.00 | 0.00 | 0.02 | 0.00 | 0.00 | 0.00 | 0.00 | 0.00 | 0.00 | 0.00 (± 0.00) |
|  | 114 | 0.00 | 0.04 | 0.00 | 0.00 | 0.00 | 0.00 | 0.00 | 0.00 | 0.00 | 0.00 | 0.00 | 0.00 | 0.00 | 0.00 | 0.00 | 0.00 (± 0.00) |
|  | **123** | **0.24** | **0.10** | **0.11** | **0.02** | **0.00** | **0.37** | **0.31** | **0.33** | **0.28** | **0.05** | **0.38** | **0.20** | **0.21** | **0.24** | **0.02** | **0.19 (± 0.03)** |
|  | 126 | 0.04 | 0.00 | 0.11 | 0.05 | 0.00 | 0.00 | 0.06 | 0.15 | 0.02 | 0.05 | 0.04 | 0.07 | 0.20 | 0.18 | 0.05 | 0.07 (± 0.02) |
|  | 129 | 0.00 | 0.00 | 0.00 | 0.02 | 0.00 | 0.00 | 0.00 | 0.00 | 0.02 | 0.00 | 0.00 | 0.00 | 0.00 | 0.02 | 0.00 | 0.00 (± 0.00) |
|  | 132 | 0.24 | 0.10 | 0.29 | 0.10 | 0.02 | 0.39 | 0.41 | 0.37 | 0.28 | 0.11 | 0.30 | 0.27 | 0.43 | 0.44 | 0.16 | 0.26 (± 0.03) |
|  | 138 | 0.12 | 0.15 | 0.07 | 0.14 | 0.07 | 0.07 | 0.03 | 0.02 | 0.10 | 0.11 | 0.06 | 0.10 | 0.02 | 0.00 | 0.16 | 0.08 (± 0.01) |
|  | 141 | 0.06 | 0.08 | 0.05 | 0.10 | 0.20 | 0.00 | 0.06 | 0.06 | 0.02 | 0.08 | 0.10 | 0.03 | 0.02 | 0.00 | 0.09 | 0.06 (± 0.01) |
|  | 144 | 0.00 | 0.00 | 0.00 | 0.00 | 0.02 | 0.00 | 0.00 | 0.00 | 0.02 | 0.00 | 0.00 | 0.00 | 0.00 | 0.00 | 0.00 | 0.00 (± 0.00) |
|  | 147 | 0.22 | 0.17 | 0.21 | 0.31 | 0.20 | 0.13 | 0.00 | 0.06 | 0.14 | 0.34 | 0.08 | 0.13 | 0.13 | 0.02 | 0.36 | 0.17 (± 0.03) |
|  | **150** | **0.08** | **0.35** | **0.16** | **0.24** | **0.43** | **0.02** | **0.13** | **0.02** | **0.10** | **0.26** | **0.04** | **0.20** | **0.00** | **0.10** | **0.16** | **0.15 (± 0.03)** |
|  | 153 | 0.00 | 0.00 | 0.00 | 0.00 | 0.05 | 0.02 | 0.00 | 0.00 | 0.02 | 0.00 | 0.00 | 0.00 | 0.00 | 0.00 | 0.00 | 0.00 (± 0.00) |
|  |  |  |  |  |  |  |  |  |  |  |  |  |  |  |  |  |  |
| DVEX03 | 187 | 0.00 | 0.02 | 0.00 | 0.00 | 0.00 | 0.00 | 0.00 | 0.00 | 0.00 | 0.00 | 0.00 | 0.00 | 0.00 | 0.00 | 0.00 | 0.00 (± 0.00) |
|  | 214 | 0.02 | 0.07 | 0.03 | 0.03 | 0.02 | 0.04 | 0.07 | 0.00 | 0.00 | 0.05 | 0.00 | 0.08 | 0.00 | 0.06 | 0.02 | 0.03 (± 0.01) |
|  | 217 | 0.00 | 0.02 | 0.00 | 0.00 | 0.00 | 0.00 | 0.00 | 0.00 | 0.02 | 0.00 | 0.00 | 0.00 | 0.00 | 0.00 | 0.00 | 0.00 (± 0.00) |
|  | 220 | 0.00 | 0.00 | 0.00 | 0.00 | 0.00 | 0.00 | 0.00 | 0.00 | 0.00 | 0.00 | 0.00 | 0.00 | 0.00 | 0.02 | 0.05 | 0.00 (± 0.00) |
|  | 223 | 0.00 | 0.00 | 0.00 | 0.00 | 0.00 | 0.00 | 0.00 | 0.00 | 0.00 | 0.00 | 0.04 | 0.00 | 0.00 | 0.00 | 0.00 | 0.00 (± 0.00) |
|  | 226 | 0.69 | 0.78 | 0.78 | 0.78 | 0.92 | 0.72 | 0.60 | 0.50 | 0.44 | 0.61 | 0.52 | 0.69 | 0.75 | 0.56 | 0.57 | 0.66 (± 0.03) |
|  | 229 | 0.04 | 0.07 | 0.02 | 0.09 | 0.04 | 0.06 | 0.02 | 0.02 | 0.22 | 0.07 | 0.02 | 0.08 | 0.06 | 0.06 | 0.07 | 0.06 (± 0.01) |
|  | 232 | 0.19 | 0.04 | 0.17 | 0.10 | 0.02 | 0.17 | 0.31 | 0.48 | 0.31 | 0.27 | 0.42 | 0.14 | 0.19 | 0.29 | 0.27 | 0.23 (± 0.03) |
|  | 235 | 0.04 | 0.00 | 0.00 | 0.00 | 0.00 | 0.00 | 0.00 | 0.00 | 0.00 | 0.00 | 0.00 | 0.00 | 0.00 | 0.00 | 0.02 | 0.00 (± 0.00) |
|  | 238 | 0.02 | 0.00 | 0.00 | 0.00 | 0.00 | 0.02 | 0.00 | 0.00 | 0.00 | 0.00 | 0.00 | 0.00 | 0.00 | 0.02 | 0.00 | 0.00 (± 0.00) |
|  |  |  |  |  |  |  |  |  |  |  |  |  |  |  |  |  |  |

Table S4. Allele frequencies for each loci, within each population for the polyploid summer dataset. Bold values denote alleles with different frequencies to those obtained from the diploid dataset (Table S3). Bold values with no background colouration represent allele frequencies that have decreased in comparison to the diploid dataset and those with grey backgrounds represent allele frequencies that have increased. Rare alleles, not present within the diploid dataset are indicated in italics.

| **POLYPLOID_Summer** | | |  |  |  |  |  |  |  |  |  |  |  |  |  |  |  |
| --- | --- | --- | --- | --- | --- | --- | --- | --- | --- | --- | --- | --- | --- | --- | --- | --- | --- |
| **Locus** | **Allele** | **Goulter** | **Schnapper** | **Hikapu** | **Nydia** | **Yncyca** | **Tawero** | **Hallam** | **Forsyth** | **Melville** | **Picton** | **Shakespeare** | **Onahau** | **Ruakaka** | **Te Aroha** | **Port Nelson** | ***Average*** |
| DVEX10 | 174 | 0.00 | 0.00 | 0.00 | 0.00 | 0.00 | 0.00 | 0.00 | 0.02 | 0.00 | 0.00 | 0.00 | 0.00 | 0.00 | 0.00 | 0.00 | 0.00 (± 0.00) |
|  | **177** | **0.02** | **0.09** | **0.10** | **0.08** | **0.13** | **0.12** | **0.02** | **0.00** | **0.06** | **0.06** | **0.01** | **0.01** | **0.05** | **0.05** | **0.01** | **0.05 (± 0.01)** |
|  | 180 | 0.10 | 0.16 | 0.10 | 0.14 | 0.04 | 0.10 | 0.13 | 0.06 | 0.13 | 0.08 | 0.08 | 0.13 | 0.11 | 0.02 | 0.01 | 0.09 (± 0.01) |
|  | 183 | 0.29 | 0.22 | 0.27 | 0.25 | 0.29 | 0.26 | 0.31 | 0.40 | 0.25 | 0.19 | 0.25 | 0.32 | 0.29 | 0.24 | 0.42 | 0.28 (± 0.02) |
|  | 186 | 0.10 | 0.12 | 0.10 | 0.08 | 0.17 | 0.12 | 0.10 | 0.05 | 0.06 | 0.08 | 0.08 | 0.03 | 0.10 | 0.08 | 0.08 | 0.09 (± 0.01) |
|  | 189 | 0.00 | 0.00 | 0.00 | 0.01 | 0.00 | 0.00 | 0.00 | 0.00 | 0.00 | 0.00 | 0.00 | 0.00 | 0.00 | 0.00 | 0.02 | 0.00 (± 0.00) |
|  | **192** | **0.33** | **0.39** | **0.41** | **0.43** | **0.36** | **0.33** | **0.43** | **0.44** | **0.50** | **0.55** | **0.54** | **0.50** | **0.40** | **0.52** | **0.43** | **0.44 (± 0.02)** |
|  | **195** | **0.16** | **0.02** | **0.02** | **0.01** | **0.02** | **0.07** | **0.01** | **0.04** | **0.00** | **0.04** | **0.03** | **0.01** | **0.05** | **0.08** | **0.02** | **0.04 (± 0.01)** |
|  |  |  |  |  |  |  |  |  |  |  |  |  |  |  |  |  |  |
| DVEX01 | 106 | 0.32 | 0.41 | 0.23 | 0.27 | 0.38 | 0.35 | 0.50 | 0.48 | 0.33 | 0.44 | 0.48 | 0.47 | 0.50 | 0.40 | 0.43 | 0.40 (± 0.02) |
|  | **112** | **0.68** | **0.59** | **0.77** | **0.71** | **0.62** | **0.65** | **0.50** | **0.48** | **0.66** | **0.56** | **0.52** | **0.53** | **0.50** | **0.60** | **0.58** | **0.60 (± 0.02)** |
|  | 115 | 0.00 | 0.00 | 0.00 | 0.00 | 0.00 | 0.00 | 0.00 | 0.04 | 0.00 | 0.00 | 0.00 | 0.00 | 0.00 | 0.00 | 0.00 | 0.00 (± 0.00) |
|  | 118 | 0.00 | 0.00 | 0.00 | 0.02 | 0.00 | 0.00 | 0.00 | 0.00 | 0.02 | 0.00 | 0.00 | 0.00 | 0.00 | 0.00 | 0.00 | 0.00 (± 0.00) |
|  |  |  |  |  |  |  |  |  |  |  |  |  |  |  |  |  |  |
| DVEX19 | 235 | 0.00 | 0.00 | 0.00 | 0.00 | 0.00 | 0.00 | 0.00 | 0.04 | 0.00 | 0.00 | 0.00 | 0.00 | 0.00 | 0.00 | 0.00 | 0.00 (± 0.00) |
|  | 239 | 0.10 | 0.02 | 0.00 | 0.00 | 0.04 | 0.11 | 0.02 | 0.06 | 0.09 | 0.03 | 0.21 | 0.28 | 0.07 | 0.09 | 0.12 | 0.08 (± 0.02) |
|  | 243 | 0.54 | 0.40 | 0.24 | 0.21 | 0.35 | 0.35 | 0.42 | 0.52 | 0.33 | 0.31 | 0.44 | 0.06 | 0.50 | 0.48 | 0.35 | 0.37 (± 0.03) |
|  | 247 | 0.10 | 0.21 | 0.56 | 0.57 | 0.29 | 0.04 | 0.33 | 0.08 | 0.17 | 0.09 | 0.08 | 0.06 | 0.00 | 0.04 | 0.03 | 0.18 (± 0.05) |
|  | 251 | 0.08 | 0.08 | 0.15 | 0.19 | 0.04 | 0.13 | 0.13 | 0.19 | 0.17 | 0.13 | 0.19 | 0.50 | 0.27 | 0.22 | 0.29 | 0.18 (± 0.03) |
|  | 255 | 0.18 | 0.21 | 0.04 | 0.03 | 0.12 | 0.28 | 0.03 | 0.10 | 0.24 | 0.16 | 0.06 | 0.00 | 0.13 | 0.11 | 0.00 | 0.11 (± 0.02) |
|  | 259 | 0.00 | 0.00 | 0.00 | 0.00 | 0.04 | 0.04 | 0.00 | 0.00 | 0.00 | 0.00 | 0.00 | 0.00 | 0.00 | 0.00 | 0.00 | 0.01 (± 0.00) |
|  | 263 | 0.00 | 0.04 | 0.02 | 0.00 | 0.13 | 0.04 | 0.07 | 0.02 | 0.00 | 0.28 | 0.02 | 0.11 | 0.04 | 0.02 | 0.21 | 0.07 (± 0.02) |
|  | 267 | 0.00 | 0.04 | 0.00 | 0.00 | 0.00 | 0.02 | 0.00 | 0.00 | 0.00 | 0.00 | 0.00 | 0.00 | 0.00 | 0.04 | 0.00 | 0.01 (± 0.00) |
|  |  |  |  |  |  |  |  |  |  |  |  |  |  |  |  |  |  |
| DVEX11 | 179 | 0.00 | 0.00 | 0.00 | 0.00 | 0.00 | 0.02 | 0.00 | 0.00 | 0.00 | 0.00 | 0.00 | 0.00 | 0.00 | 0.00 | 0.00 | 0.00 (± 0.00) |
|  | *185* | *0.00* | *0.00* | *0.00* | *0.00* | *0.00* | *0.00* | *0.00* | *0.00* | *0.01* | *0.00* | *0.00* | *0.00* | *0.00* | *0.00* | *0.00* | *0.00 (± 0.00)* |
|  | 195 | 0.04 | 0.08 | 0.07 | 0.14 | 0.01 | 0.13 | 0.04 | 0.03 | 0.01 | 0.02 | 0.05 | 0.06 | 0.02 | 0.06 | 0.06 | 0.06 (± 0.01) |
|  | **197** | **0.10** | **0.13** | **0.07** | **0.06** | **0.13** | **0.02** | **0.07** | **0.04** | **0.07** | **0.08** | **0.07** | **0.05** | **0.01** | **0.03** | **0.27** | **0.08 (± 0.02)** |
|  | *199* | *0.00* | *0.00* | *0.00* | *0.00* | *0.00* | *0.00* | *0.00* | *0.01* | *0.00* | *0.00* | *0.00* | *0.00* | *0.00* | *0.00* | *0.00* | *0.00 (± 0.00)* |
|  | 201 | 0.00 | 0.00 | 0.00 | 0.00 | 0.00 | 0.00 | 0.00 | 0.00 | 0.00 | 0.02 | 0.00 | 0.00 | 0.00 | 0.00 | 0.00 | 0.00 (± 0.00) |
|  | 205 | 0.08 | 0.03 | 0.04 | 0.02 | 0.06 | 0.02 | 0.02 | 0.08 | 0.06 | 0.04 | 0.06 | 0.00 | 0.17 | 0.08 | 0.00 | 0.05 (± 0.01) |
|  | **207** | **0.27** | **0.43** | **0.64** | **0.50** | **0.42** | **0.57** | **0.54** | **0.36** | **0.41** | **0.49** | **0.26** | **0.66** | **0.27** | **0.26** | **0.50** | **0.44 (± 0.03)** |
|  | 213 | 0.00 | 0.00 | 0.00 | 0.00 | 0.02 | 0.00 | 0.00 | 0.00 | 0.00 | 0.00 | 0.00 | 0.00 | 0.00 | 0.00 | 0.00 | 0.00 (± 0.00) |
|  | 215 | 0.04 | 0.00 | 0.00 | 0.00 | 0.00 | 0.00 | 0.00 | 0.08 | 0.00 | 0.00 | 0.02 | 0.00 | 0.00 | 0.00 | 0.05 | 0.01 (± 0.01) |
|  | **217** | **0.48** | **0.35** | **0.18** | **0.28** | **0.36** | **0.24** | **0.33** | **0.40** | **0.44** | **0.34** | **0.54** | **0.24** | **0.53** | **0.56** | **0.12** | **0.36 (± 0.03)** |
|  |  |  |  |  |  |  |  |  |  |  |  |  |  |  |  |  |  |
| DVEX36 | 142 | 0.00 | 0.00 | 0.00 | 0.00 | 0.00 | 0.00 | 0.00 | 0.01 | 0.00 | 0.00 | 0.00 | 0.07 | 0.00 | 0.00 | 0.00 | 0.01 (± 0.00) |
|  | 151 | 0.00 | 0.00 | 0.00 | 0.02 | 0.00 | 0.00 | 0.00 | 0.00 | 0.00 | 0.00 | 0.00 | 0.00 | 0.01 | 0.00 | 0.00 | 0.00 (± 0.00) |
|  | 154 | 0.00 | 0.00 | 0.00 | 0.00 | 0.00 | 0.00 | 0.00 | 0.01 | 0.04 | 0.01 | 0.00 | 0.00 | 0.00 | 0.00 | 0.00 | 0.00 (± 0.00) |
|  | 163 | 0.00 | 0.00 | 0.00 | 0.00 | 0.00 | 0.00 | 0.00 | 0.00 | 0.02 | 0.01 | 0.00 | 0.00 | 0.00 | 0.00 | 0.00 | 0.00 (± 0.00) |
|  | 166 | 0.25 | 0.31 | 0.34 | 0.31 | 0.19 | 0.37 | 0.21 | 0.20 | 0.24 | 0.21 | 0.29 | 0.20 | 0.14 | 0.26 | 0.23 | 0.25 (± 0.02) |
|  | 169 | 0.02 | 0.08 | 0.13 | 0.10 | 0.10 | 0.06 | 0.12 | 0.07 | 0.02 | 0.02 | 0.08 | 0.08 | 0.07 | 0.13 | 0.10 | 0.08 (± 0.01) |
|  | 172 | 0.00 | 0.00 | 0.00 | 0.00 | 0.00 | 0.00 | 0.00 | 0.00 | 0.00 | 0.00 | 0.02 | 0.00 | 0.02 | 0.00 | 0.00 | 0.00 (± 0.00) |
|  | 175 | 0.62 | 0.58 | 0.54 | 0.57 | 0.71 | 0.56 | 0.67 | 0.70 | 0.68 | 0.68 | 0.62 | 0.65 | 0.73 | 0.61 | 0.60 | 0.63 (± 0.02) |
|  | 178 | 0.12 | 0.02 | 0.00 | 0.00 | 0.00 | 0.02 | 0.00 | 0.00 | 0.00 | 0.06 | 0.00 | 0.00 | 0.04 | 0.00 | 0.06 | 0.02 (± 0.01) |
|  |  |  |  |  |  |  |  |  |  |  |  |  |  |  |  |  |  |
| DVEX30 | 211 | 0.00 | 0.00 | 0.00 | 0.00 | 0.00 | 0.00 | 0.00 | 0.00 | 0.04 | 0.00 | 0.00 | 0.00 | 0.00 | 0.00 | 0.00 | 0.00 (± 0.00) |
|  | 214 | 0.00 | 0.05 | 0.00 | 0.08 | 0.00 | 0.00 | 0.06 | 0.00 | 0.02 | 0.00 | 0.06 | 0.00 | 0.00 | 0.00 | 0.00 | 0.02 (± 0.01) |
|  | 229 | 0.00 | 0.00 | 0.00 | 0.00 | 0.00 | 0.00 | 0.00 | 0.00 | 0.00 | 0.00 | 0.00 | 0.00 | 0.00 | 0.00 | 0.02 | 0.00 (± 0.00) |
|  | 232 | 0.02 | 0.00 | 0.00 | 0.00 | 0.00 | 0.00 | 0.00 | 0.00 | 0.00 | 0.00 | 0.01 | 0.00 | 0.00 | 0.00 | 0.00 | 0.00 (± 0.00) |
|  | 235 | 0.02 | 0.03 | 0.02 | 0.06 | 0.00 | 0.00 | 0.00 | 0.00 | 0.05 | 0.00 | 0.00 | 0.00 | 0.04 | 0.02 | 0.00 | 0.02 (± 0.01) |
|  | 238 | 0.12 | 0.28 | 0.29 | 0.22 | 0.13 | 0.28 | 0.09 | 0.13 | 0.14 | 0.00 | 0.09 | 0.06 | 0.12 | 0.12 | 0.02 | 0.14 (± 0.02) |
|  | 241 | 0.21 | 0.03 | 0.02 | 0.00 | 0.02 | 0.14 | 0.03 | 0.03 | 0.00 | 0.40 | 0.22 | 0.22 | 0.07 | 0.08 | 0.53 | 0.13 (± 0.04) |
|  | 244 | 0.00 | 0.00 | 0.02 | 0.00 | 0.00 | 0.00 | 0.00 | 0.01 | 0.04 | 0.00 | 0.00 | 0.06 | 0.00 | 0.00 | 0.00 | 0.01 (± 0.00) |
|  | 247 | 0.03 | 0.08 | 0.00 | 0.00 | 0.00 | 0.00 | 0.00 | 0.02 | 0.10 | 0.02 | 0.02 | 0.00 | 0.00 | 0.00 | 0.01 | 0.02 (± 0.01) |
|  | 250 | 0.56 | 0.44 | 0.54 | 0.53 | 0.56 | 0.55 | 0.78 | 0.81 | 0.39 | 0.58 | 0.53 | 0.67 | 0.76 | 0.75 | 0.41 | 0.59 (± 0.03) |
|  | 253 | 0.04 | 0.00 | 0.00 | 0.00 | 0.04 | 0.03 | 0.00 | 0.00 | 0.05 | 0.00 | 0.07 | 0.00 | 0.00 | 0.04 | 0.00 | 0.02 (± 0.01) |
|  | 256 | 0.00 | 0.00 | 0.00 | 0.01 | 0.00 | 0.00 | 0.00 | 0.00 | 0.02 | 0.00 | 0.00 | 0.00 | 0.00 | 0.00 | 0.00 | 0.00 (± 0.00) |
|  | 259 | 0.00 | 0.10 | 0.11 | 0.10 | 0.25 | 0.00 | 0.03 | 0.00 | 0.15 | 0.00 | 0.00 | 0.00 | 0.02 | 0.00 | 0.00 | 0.05 (± 0.02) |
|  |  |  |  |  |  |  |  |  |  |  |  |  |  |  |  |  |  |
| DVEX33 | 105 | 0.00 | 0.00 | 0.00 | 0.01 | 0.00 | 0.00 | 0.00 | 0.00 | 0.00 | 0.00 | 0.00 | 0.00 | 0.00 | 0.00 | 0.00 | 0.00 (± 0.00) |
|  | 111 | 0.00 | 0.00 | 0.00 | 0.00 | 0.00 | 0.00 | 0.00 | 0.00 | 0.01 | 0.00 | 0.00 | 0.00 | 0.00 | 0.00 | 0.00 | 0.00 (± 0.00) |
|  | 114 | 0.00 | 0.04 | 0.00 | 0.01 | 0.00 | 0.00 | 0.00 | 0.00 | 0.00 | 0.00 | 0.00 | 0.00 | 0.00 | 0.00 | 0.00 | 0.00 (± 0.00) |
|  | **123** | **0.20** | **0.10** | **0.09** | **0.02** | **0.00** | **0.34** | **0.26** | **0.31** | **0.25** | **0.05** | **0.35** | **0.18** | **0.20** | **0.23** | **0.02** | **0.17 (± 0.03)** |
|  | 126 | 0.03 | 0.00 | 0.11 | 0.05 | 0.00 | 0.00 | 0.05 | 0.15 | 0.01 | 0.04 | 0.03 | 0.04 | 0.18 | 0.17 | 0.05 | 0.06 (± 0.02) |
|  | 129 | 0.00 | 0.00 | 0.00 | 0.01 | 0.00 | 0.00 | 0.04 | 0.00 | 0.01 | 0.00 | 0.00 | 0.00 | 0.00 | 0.01 | 0.00 | 0.01 (± 0.00) |
|  | 132 | 0.26 | 0.10 | 0.28 | 0.10 | 0.02 | 0.36 | 0.33 | 0.35 | 0.29 | 0.10 | 0.26 | 0.22 | 0.41 | 0.45 | 0.15 | 0.25 (± 0.03) |
|  | *135* | *0.00* | *0.00* | *0.00* | *0.00* | *0.00* | *0.00* | *0.02* | *0.01* | *0.00* | *0.00* | *0.00* | *0.00* | *0.00* | *0.01* | *0.00* | *0.00 (± 0.00)* |
|  | 138 | 0.13 | 0.14 | 0.07 | 0.13 | 0.07 | 0.08 | 0.02 | 0.02 | 0.10 | 0.11 | 0.05 | 0.10 | 0.02 | 0.00 | 0.16 | 0.08 (± 0.01) |
|  | 141 | 0.04 | 0.08 | 0.05 | 0.10 | 0.20 | 0.01 | 0.07 | 0.05 | 0.03 | 0.08 | 0.09 | 0.03 | 0.02 | 0.00 | 0.09 | 0.06 (± 0.01) |
|  | 144 | 0.00 | 0.00 | 0.00 | 0.00 | 0.02 | 0.00 | 0.00 | 0.01 | 0.02 | 0.00 | 0.00 | 0.00 | 0.00 | 0.00 | 0.00 | 0.00 (± 0.00) |
|  | 147 | 0.20 | 0.17 | 0.23 | 0.30 | 0.20 | 0.13 | 0.00 | 0.06 | 0.13 | 0.36 | 0.09 | 0.13 | 0.12 | 0.01 | 0.38 | 0.17 (± 0.03) |
|  | **150** | **0.13** | **0.37** | **0.16** | **0.26** | **0.43** | **0.06** | **0.22** | **0.06** | **0.13** | **0.26** | **0.12** | **0.29** | **0.06** | **0.11** | **0.16** | **0.19 (± 0.03)** |
|  | 153 | 0.00 | 0.00 | 0.00 | 0.00 | 0.05 | 0.02 | 0.00 | 0.00 | 0.02 | 0.00 | 0.00 | 0.00 | 0.00 | 0.00 | 0.00 | 0.01 (± 0.00) |
|  |  |  |  |  |  |  |  |  |  |  |  |  |  |  |  |  |  |
| DVEX03 | 187 | 0.00 | 0.02 | 0.00 | 0.00 | 0.00 | 0.00 | 0.00 | 0.00 | 0.00 | 0.00 | 0.00 | 0.00 | 0.00 | 0.00 | 0.00 | 0.00 (± 0.00) |
|  | 214 | 0.02 | 0.07 | 0.03 | 0.03 | 0.02 | 0.04 | 0.07 | 0.00 | 0.00 | 0.05 | 0.00 | 0.08 | 0.00 | 0.06 | 0.02 | 0.03 (± 0.01) |
|  | 217 | 0.00 | 0.02 | 0.00 | 0.00 | 0.00 | 0.00 | 0.00 | 0.00 | 0.02 | 0.00 | 0.00 | 0.00 | 0.00 | 0.00 | 0.00 | 0.00 (± 0.00) |
|  | 220 | 0.00 | 0.00 | 0.00 | 0.00 | 0.00 | 0.00 | 0.00 | 0.00 | 0.00 | 0.00 | 0.00 | 0.00 | 0.00 | 0.02 | 0.05 | 0.00 (± 0.00) |
|  | 223 | 0.00 | 0.00 | 0.00 | 0.00 | 0.00 | 0.00 | 0.00 | 0.00 | 0.00 | 0.00 | 0.04 | 0.00 | 0.00 | 0.00 | 0.00 | 0.00 (± 0.00) |
|  | 226 | 0.69 | 0.78 | 0.78 | 0.78 | 0.92 | 0.72 | 0.60 | 0.50 | 0.44 | 0.61 | 0.52 | 0.69 | 0.75 | 0.56 | 0.57 | 0.66 (± 0.03) |
|  | 229 | 0.04 | 0.07 | 0.02 | 0.09 | 0.04 | 0.06 | 0.02 | 0.02 | 0.22 | 0.07 | 0.02 | 0.08 | 0.06 | 0.06 | 0.07 | 0.06 (± 0.01) |
|  | 232 | 0.19 | 0.04 | 0.17 | 0.10 | 0.02 | 0.17 | 0.31 | 0.48 | 0.31 | 0.27 | 0.42 | 0.14 | 0.19 | 0.29 | 0.27 | 0.23 (± 0.03) |
|  | 235 | 0.04 | 0.00 | 0.00 | 0.00 | 0.00 | 0.00 | 0.00 | 0.00 | 0.00 | 0.00 | 0.00 | 0.00 | 0.00 | 0.00 | 0.02 | 0.00 (± 0.00) |
|  | 238 | 0.02 | 0.00 | 0.00 | 0.00 | 0.00 | 0.02 | 0.00 | 0.00 | 0.00 | 0.00 | 0.00 | 0.00 | 0.00 | 0.02 | 0.00 | 0.00 (± 0.00) |
|  |  |  |  |  |  |  |  |  |  |  |  |  |  |  |  |  |  |

Table S5. Pairwise FST (below diagonal) and Jost’s D (above diagonal) matrix (including all loci) for each cluster grouping for winter populations for Pelorus Sound, from 24 hours (a), to 12 hours (b) and 2 hours (c) for the winter Pelorus Sound samples. Significant pairwise combinations after False Discovery Rate corrections for multiple tests (N=15) are indicated in bold, P ≤ 0.003. Pelorus Sound populations include: GOUL=Goulter Bay, HIKA=Hikapu Reach, YNCY=Yncyca Bay, HALM= Hallam Cove, FORS=Forsyth Bay and MELV=Melville Cove.

| **24-hour PLD** | |  |  |  |  |  |  |
| --- | --- | --- | --- | --- | --- | --- | --- |
| *Clust 1* | | *Clust 2* | | | | *Clust 3* |  |
|  | GOUL | HIKA | YNCY | HALM | FORS | MELV |  |
| GOUL | - | **0.00** | **0.10** | **0.04** | **0.07** | **0.10** |  |
| HIKA | **0.09** | - | **0.04** | **0.16** | **0.18** | **0.19** |  |
| YNCY | **0.04** | 0.03 | - | **0.14** | **0.17** | **0.14** |  |
| HALM | 0.02 | **0.08** | **0.06** | - | 0.02 | **0.06** |  |
| FORS | **0.03** | **0.10** | **0.07** | 0.01 | - | **0.07** |  |
| MELV | **0.06** | **0.12** | **0.10** | **0.03** | **0.05** | - |  |
|  |  |  |  |  |  |  |  |
|  |  |  |  |  |  |  |  |
|  |  |  |  |  |  |  |  |
| **12-hour PLD** | |  |  |  |  |  |  |
| *Clust 1* | | *Clust 2* | | | *Clust 3* | *Clust 4* |  |
|  | GOUL | HIKA | YNCY | FORS | HALM | MELV |  |
| GOUL | - | **0.00** | **0.10** | **0.07** | **0.04** | **0.10** |  |
| HIKA | **0.09** | - | **0.04** | **0.18** | **0.16** | **0.19** |  |
| YNCY | **0.04** | 0.03 | - | **0.17** | **0.14** | **0.14** |  |
| FORS | **0.03** | **0.10** | **0.07** | - | 0.02 | **0.07** |  |
| HALM | 0.02 | **0.08** | **0.06** | 0.01 | - | **0.06** |  |
| MELV | **0.06** | **0.12** | **0.10** | **0.05** | **0.03** | - |  |
|  |  |  |  |  |  |  |  |
|  |  |  |  |  |  |  |  |
| **2-hour PLD** | |  |  |  |  |  |  |
| *Clust 1* | | *Clust 3* | *Cluster 5* | *Clust 7* | *Clust 8* | *Clust 9* |  |
|  | GOUL | HIKA | YNCY | FORS | HALM | MELV |  |
| GOUL | - | **0.00** | **0.10** | **0.07** | **0.04** | **0.10** |  |
| HIKA | **0.09** | - | **0.04** | **0.18** | **0.16** | **0.19** |  |
| YNCY | **0.04** | 0.03 | - | **0.17** | **0.14** | **0.14** |  |
| FORS | **0.03** | **0.10** | **0.07** | - | 0.02 | **0.07** |  |
| HALM | 0.02 | **0.08** | **0.06** | 0.01 | - | **0.06** |  |
| MELV | **0.06** | **0.12** | **0.10** | **0.05** | **0.03** | - |  |
|  |  |  |  |  |  |  |  |

Table S6 Allele frequencies for each loci within each population for the diploid winter dataset. Bold values denote alleles with different frequencies to those attained using the polyploid dataset (polyploid winter dataset found in Table S7).

| **DIPLOID_winter** | |  |  |  |  |  |  |  |
| --- | --- | --- | --- | --- | --- | --- | --- | --- |
| **Locus** | **Allele** | **Goulter** | **Hikapu** | **Yncyca** | **Hallam Cove** | **Forsyth** | **Melville** | **Average** |
| DVEX10 | **177** | **0.00** | **0.07** | **0.05** | **0.00** | **0.02** | **0.00** | **0.02 (± 0.01)** |
|  | 180 | 0.00 | 0.07 | 0.02 | 0.00 | 0.04 | 0.09 | 0.04 (± 0.02) |
|  | **183** | **0.24** | **0.24** | **0.20** | **0.21** | **0.31** | **0.23** | **0.24 (± 0.10)** |
|  | 186 | 0.11 | 0.19 | 0.30 | 0.12 | 0.06 | 0.02 | 0.13 (± 0.05) |
|  | **192** | **0.46** | **0.43** | **0.43** | **0.48** | **0.48** | **0.59** | **0.48 (± 0.19)** |
|  | **195** | **0.20** | **0.00** | **0.00** | **0.19** | **0.08** | **0.07** | **0.09 (± 0.04)** |
| DVEX01 | 100 | 0.00 | 0.00 | 0.00 | 0.00 | 0.02 | 0.00 | 0.00 (± 0.00) |
|  | 106 | 0.42 | 0.36 | 0.12 | 0.45 | 0.38 | 0.30 | 0.34 (± 0.14) |
|  | 112 | 0.58 | 0.64 | 0.88 | 0.55 | 0.60 | 0.70 | 0.66 (± 0.27) |
| DVEX19 | 239 | 0.27 | 0.16 | 0.05 | 0.33 | 0.58 | 0.25 | 0.27 (± 0.11) |
|  | 243 | 0.08 | 0.06 | 0.15 | 0.10 | 0.02 | 0.06 | 0.08 (± 0.03) |
|  | 247 | 0.04 | 0.50 | 0.35 | 0.17 | 0.06 | 0.09 | 0.20 (± 0.08) |
|  | **251** | **0.60** | **0.28** | **0.45** | **0.21** | **0.21** | **0.56** | **0.39 (± 0.16)** |
|  | 255 | 0.00 | 0.00 | 0.00 | 0.05 | 0.10 | 0.00 | 0.03 (± 0.01) |
|  | 259 | 0.00 | 0.00 | 0.00 | 0.10 | 0.00 | 0.00 | 0.02 (± 0.01) |
|  | 263 | 0.02 | 0.00 | 0.00 | 0.05 | 0.00 | 0.03 | 0.02 (± 0.01) |
|  | 267 | 0.00 | 0.00 | 0.00 | 0.00 | 0.02 | 0.00 | 0.00 (± 0.00) |
| DVEX11 | 173 | 0.02 | 0.00 | 0.00 | 0.00 | 0.00 | 0.00 | 0.00 (± 0.00) |
|  | **195** | **0.09** | **0.03** | **0.00** | **0.02** | **0.07** | **0.00** | **0.04 (± 0.01)** |
|  | 199 | 0.00 | 0.00 | 0.00 | 0.00 | 0.02 | 0.00 | 0.00 (± 0.00) |
|  | 205 | 0.06 | 0.00 | 0.00 | 0.12 | 0.11 | 0.03 | 0.05 (± 0.02) |
|  | **207** | **0.33** | **0.84** | **0.60** | **0.40** | **0.28** | **0.40** | **0.48 (± 0.19)** |
| DVEX36 | 160 | 0.02 | 0.00 | 0.00 | 0.02 | 0.00 | 0.00 | 0.01 (± 0.00) |
|  | 166 | 0.17 | 0.24 | 0.19 | 0.15 | 0.33 | 0.05 | 0.19 (± 0.08) |
|  | **169** | **0.17** | **0.03** | **0.00** | **0.13** | **0.04** | **0.02** | **0.06 (± 0.03)** |
|  | 172 | 0.02 | 0.00 | 0.00 | 0.02 | 0.00 | 0.00 | 0.01 (± 0.00) |
|  | 175 | 0.30 | 0.68 | 0.60 | 0.46 | 0.52 | 0.67 | 0.54 (± 0.22) |
|  | 178 | 0.33 | 0.03 | 0.21 | 0.23 | 0.11 | 0.26 | 0.20 (± 0.08) |
|  | **187** | **0.00** | **0.03** | **0.00** | **0.00** | **0.00** | **0.00** | **0.00 (± 0.00)** |
| DVEX30 | 226 | 0.00 | 0.00 | 0.00 | 0.13 | 0.00 | 0.00 | 0.02 (± 0.01) |
|  | 235 | 0.02 | 0.00 | 0.00 | 0.13 | 0.00 | 0.03 | 0.03 (± 0.01) |
|  | 238 | 0.22 | 0.03 | 0.00 | 0.07 | 0.22 | 0.00 | 0.09 (± 0.04) |
|  | 241 | 0.22 | 0.17 | 0.03 | 0.09 | 0.06 | 0.00 | 0.09 (± 0.04) |
|  | **247** | **0.00** | **0.00** | **0.00** | **0.00** | **0.04** | **0.00** | **0.01 (± 0.00)** |
|  | **250** | **0.52** | **0.81** | **0.68** | **0.54** | **0.62** | **0.87** | **0.67 (± 0.27)** |
|  | **253** | **0.00** | **0.00** | **0.03** | **0.00** | **0.02** | **0.00** | **0.01 (± 0.00)** |
| DVEX33 | 114 | 0.02 | 0.00 | 0.00 | 0.00 | 0.00 | 0.00 | 0.00 (± 0.00) |
|  | **123** | **0.13** | **0.08** | **0.08** | **0.23** | **0.33** | **0.33** | **0.20 (± 0.08)** |
|  | **126** | **0.00** | **0.00** | **0.00** | **0.04** | **0.00** | **0.05** | **0.01 (± 0.01)** |
|  | **132** | **0.26** | **0.11** | **0.16** | **0.31** | **0.38** | **0.53** | **0.29 (± 0.12)** |
|  | 138 | 0.11 | 0.18 | 0.03 | 0.08 | 0.07 | 0.00 | 0.08 (± 0.03) |
|  | 141 | 0.02 | 0.08 | 0.05 | 0.02 | 0.00 | 0.03 | 0.03 (± 0.01) |
|  | 150 | 0.33 | 0.47 | 0.42 | 0.12 | 0.17 | 0.08 | 0.26 (± 0.11) |
|  | **153** | **0.13** | **0.08** | **0.26** | **0.21** | **0.05** | **0.00** | **0.12 (± 0.05)** |
| DVEX03 | 196 | 0.00 | 0.00 | 0.00 | 0.02 | 0.00 | 0.00 | 0.00 (± 0.00) |
|  | 205 | 0.00 | 0.00 | 0.00 | 0.02 | 0.00 | 0.00 | 0.00 (± 0.00) |
|  | 208 | 0.00 | 0.02 | 0.00 | 0.00 | 0.00 | 0.00 | 0.00 (± 0.00) |
|  | 214 | 0.00 | 0.05 | 0.02 | 0.00 | 0.00 | 0.00 | 0.01 (± 0.00) |
|  | 220 | 0.02 | 0.00 | 0.00 | 0.00 | 0.00 | 0.00 | 0.00 (± 0.00) |
|  | 226 | 0.79 | 0.67 | 0.93 | 0.60 | 0.71 | 0.47 | 0.70 (± 0.28) |
|  | 229 | 0.00 | 0.12 | 0.00 | 0.02 | 0.05 | 0.14 | 0.06 (± 0.02) |
|  | 232 | 0.15 | 0.10 | 0.05 | 0.26 | 0.21 | 0.25 | 0.17 (± 0.07) |
|  | **235** | **0.04** | **0.05** | **0.00** | **0.08** | **0.02** | **0.14** | **0.05 (± 0.02)** |

Table S7. Allele frequencies for each loci, within each population for the polyploid winter dataset. Bold values denote alleles with different frequencies to those obtained from the diploid dataset (Table S6). Bold values with no background colouration represent allele frequencies that have decreased in comparison to the diploid dataset and those with grey backgrounds represent allele frequencies that have increased. Rare alleles, not present within the diploid dataset are indicated in italics.

| **POLYPLOID_winter** | |  |  |  |  |  |  |  |
| --- | --- | --- | --- | --- | --- | --- | --- | --- |
| **Locus** | **Allele** | **Goulter** | **Hikapu** | **Yncyca** | **Hallam Cove** | **Forsyth** | **Melville** | **Average** |
| **DVEX10** | **177** | **0.04** | **0.11** | **0.17** | **0.04** | **0.02** | **0.02** | **0.07 (± 0.02)** |
|  | 180 | 0.00 | 0.06 | 0.01 | 0.02 | 0.04 | 0.09 | 0.04 (± 0.01) |
|  | **183** | **0.3** | **0.3** | **0.28** | **0.24** | **0.31** | **0.24** | **0.28 (± 0.01)** |
|  | 186 | 0.12 | 0.15 | 0.20 | 0.15 | 0.08 | 0.05 | 0.13 (± 0.02) |
|  | ***189*** | **0.00** | **0.00** | ***0.01*** | **0.00** | **0.00** | **0.00** | ***0.00 (± 0.00)*** |
|  | **192** | **0.39** | **0.38** | **0.32** | **0.41** | **0.45** | **0.56** | **0.42 (± 0.03)** |
|  | **195** | **0.16** | **0.00** | **0.00** | **0.13** | **0.07** | **0.05** | **0.07 (± 0.03)** |
|  | ***198*** | ***0.00*** | ***0.00*** | ***0.00*** | ***0.00*** | ***0.02*** | 0.00 | ***0.00 (± 0.00)*** |
| DVEX01 | 100 | 0.00 | 0.00 | 0.00 | 0.00 | 0.02 | 0.00 | 0.00 (± 0.00) |
|  | 106 | 0.42 | 0.36 | 0.12 | 0.45 | 0.37 | 0.30 | 0.34 (± 0.05) |
|  | 112 | 0.58 | 0.64 | 0.88 | 0.55 | 0.59 | 0.70 | 0.66 (± 0.05) |
| DVEX19 | 239 | 0.26 | 0.16 | 0.05 | 0.33 | 0.58 | 0.25 | 0.27 (± 0.07) |
|  | 243 | 0.08 | 0.06 | 0.15 | 0.10 | 0.02 | 0.06 | 0.08 (± 0.02) |
|  | 247 | 0.04 | 0.50 | 0.35 | 0.17 | 0.06 | 0.09 | 0.20 (± 0.08) |
|  | **251** | **0.59** | **0.28** | **0.45** | **0.21** | **0.21** | **0.56** | **0.38 (± 0.07)** |
|  | 255 | 0.01 | 0.00 | 0.00 | 0.06 | 0.10 | 0.00 | 0.03 (± 0.02) |
|  | 259 | 0.00 | 0.00 | 0.00 | 0.09 | 0.00 | 0.00 | 0.02 (± 0.02) |
|  | 263 | 0.02 | 0.00 | 0.00 | 0.05 | 0.00 | 0.03 | 0.02 (± 0.01) |
|  | 267 | 0.00 | 0.00 | 0.00 | 0.00 | 0.02 | 0.00 | 0.00 (± 0.00) |
| DVEX11 | 173 | 0.02 | 0.00 | 0.00 | 0.00 | 0.00 | 0.00 | 0.00 (± 0.00) |
|  | ***191*** | ***0.00*** | ***0.00*** | ***0.00*** | ***0.00*** | ***0.09*** | ***0.00*** | ***0.02(± 0.02)*** |
|  | **195** | **0.09** | **0.03** | **0.00** | **0.02** | **0.14** | **0.00** | **0.05 (± 0.02)** |
|  | 199 | 0.00 | 0.00 | 0.00 | 0.00 | 0.01 | 0.00 | 0.00 (± 0.00) |
|  | 205 | 0.06 | 0.00 | 0.00 | 0.12 | 0.10 | 0.03 | 0.05 (± 0.02) |
|  | **207** | **0.33** | **0.84** | **0.6** | **0.40** | **0.27** | **0.40** | **0.47 (± 0.09)** |
| DVEX36 | 160 | 0.02 | 0.00 | 0.00 | 0.02 | 0.00 | 0.00 | 0.01 (± 0.00) |
|  | 166 | 0.17 | 0.24 | 0.19 | 0.15 | 0.33 | 0.05 | 0.19 (± 0.04) |
|  | **169** | **0.17** | **0.03** | **0.00** | **0.13** | **0.04** | **0.02** | **0.07 (± 0.03)** |
|  | 172 | 0.02 | 0.00 | 0.00 | 0.02 | 0.00 | 0.00 | 0.01 (± 0.00) |
|  | 175 | 0.3 | 0.68 | 0.6 | 0.46 | 0.52 | 0.67 | 0.54 (± 0.06) |
|  | 178 | 0.33 | 0.03 | 0.21 | 0.23 | 0.11 | 0.26 | 0.20 (± 0.04) |
|  | **187** | **0.00** | **0.03** | **0.00** | **0.00** | **0.00** | **0.00** | **0.01 (± 0.01)** |
| DVEX30 | 226 | 0.00 | 0.00 | 0.00 | 0.10 | 0.00 | 0.00 | 0.02 (± 0.02) |
|  | ***232*** | ***0.00*** | ***0.00*** | ***0.00*** | ***0.04*** | ***0.00*** | ***0.00*** | ***0.01 (± 0.01)*** |
|  | 235 | 0.02 | 0.00 | 0.00 | 0.13 | 0.00 | 0.03 | 0.03 (± 0.02) |
|  | 238 | 0.2 | 0.03 | 0.00 | 0.06 | 0.22 | 0.00 | 0.09 (± 0.04) |
|  | 241 | 0.2 | 0.17 | 0.03 | 0.09 | 0.04 | 0.00 | 0.09 (± 0.03) |
|  | **247** | **0.00** | **0.00** | **0.00** | **0.00** | **0.02** | **0.00** | **0.00 (± 0.00)** |
|  | **250** | **0.56** | **0.81** | **0.68** | **0.54** | **0.63** | **0.87** | **0.68 (± 0.05)** |
|  | **253** | 0.00 | 0.00 | **0.03** | **0.03** | **0.04** | 0.00 | **0.02 (± 0.01)** |
| DVEX33 | 114 | 0.01 | 0.00 | 0.00 | 0.00 | 0.00 | 0.00 | 0.00 (± 0.00) |
|  | **123** | **0.12** | **0.07** | **0.08** | **0.22** | **0.31** | **0.30** | **0.18 (± 0.04)** |
|  | **126** | 0.00 | 0.00 | 0.00 | **0.04** | 0.00 | **0.05** | **0.02 (± 0.01)** |
|  | **132** | **0.26** | **0.10** | **0.16** | **0.3** | **0.35** | **0.49** | **0.28 (± 0.06)** |
|  | 138 | 0.11 | 0.18 | 0.03 | 0.08 | 0.07 | 0.00 | 0.08 (± 0.03) |
|  | 141 | 0.02 | 0.08 | 0.05 | 0.02 | 0.00 | 0.02 | 0.03 (± 0.01) |
|  | ***147*** | ***0.01*** | ***0.00*** | ***0.00*** | ***0.01*** | ***0.04*** | ***0.06*** | ***0.02 (± 0.01)*** |
|  | 150 | 0.33 | 0.46 | 0.42 | 0.12 | 0.17 | 0.07 | 0.26 (± 0.07) |
|  | **153** | **0.13** | **0.12** | **0.26** | **0.21** | **0.06** | **0.06** | **0.14 (± 0.03)** |
| DVEX03 | 196 | 0.00 | 0.00 | 0.00 | 0.02 | 0.00 | 0.00 | 0.00 (± 0.00) |
|  | 205 | 0.00 | 0.00 | 0.00 | 0.02 | 0.00 | 0.00 | 0.00 (± 0.00) |
|  | 208 | 0.00 | 0.02 | 0.00 | 0.00 | 0.00 | 0.00 | 0.00 (± 0.00) |
|  | 214 | 0.00 | 0.05 | 0.02 | 0.00 | 0.00 | 0.00 | 0.01 (± 0.01) |
|  | 220 | 0.02 | 0.00 | 0.00 | 0.00 | 0.00 | 0.00 | 0.00 (± 0.00) |
|  | 226 | 0.79 | 0.67 | 0.93 | 0.6 | 0.71 | 0.47 | 0.70 (± 0.06) |
|  | 229 | 0.00 | 0.12 | 0.00 | 0.02 | 0.05 | 0.14 | 0.06 (± 0.03) |
|  | 232 | 0.15 | 0.10 | 0.05 | 0.26 | 0.21 | 0.25 | 0.17 (± 0.03) |
|  | **235** | **0.05** | **0.06** | **0** | **0.09** | **0.04** | **0.16** | **0.07 (± 0.02)** |
